# Deep learning model based on multi-lesion and time series CT images for predicting the benefits from anti-HER2 targeted therapy in stage IV gastric cancer

**DOI:** 10.1186/s13244-024-01639-2

**Published:** 2024-02-27

**Authors:** Meng He, Zi-fan Chen, Song Liu, Yang Chen, Huan Zhang, Li Zhang, Jie Zhao, Jie Yang, Xiao-tian Zhang, Lin Shen, Jian-bo Gao, Bin Dong, Lei Tang

**Affiliations:** 1 Department of Radiology, Key Laboratory of Carcinogenesis and Translational Research (Ministry of Education), Peking University Cancer Hospital and Institute, Beijing, China; 2 Center for Data Science, Peking University, Beijing, China; 3 Department of Radiology, Nanjing Drum Tower Hospital, The Affiliated Hospital of Nanjing University Medical School, No. 321, Zhongshan Road, Nanjing CityJiangsu Province, 210008 China; 4 Department of Gastrointestinal Oncology, Key Laboratory of Carcinogenesis and Translational Research (Ministry of Education/Beijing), Peking University Cancer Hospital & Institute, Beijing, China; 5 Department of Radiology, Ruijin Hospital, Shanghai Jiao Tong University School of Medicine, Shanghai, 200025 China; 6 National Engineering Laboratory for Big Data Analysis and Applications, Peking University, Beijing, China; 7 Peking University Changsha Institute for Computing and Digital Economy, Changsha, China; 8 Department of Radiology, The First Affiliated Hospital of Zhengzhou University, Zhengzhou, China; 9 Beijing International Center for Mathematical Research, Peking University, Beijing, China; 10 Center for Machine Learning Research, Peking University, Beijing, China

**Keywords:** Human epidermal growth factor receptor 2, Deep learning, Gastric cancer, Computed tomography (X-ray), Treatment outcome

## Abstract

**Objective:**

To develop and validate a deep learning model based on multi-lesion and time series CT images in predicting overall survival (OS) in patients with stage IV gastric cancer (GC) receiving anti-HER2 targeted therapy.

**Methods:**

A total of 207 patients were enrolled in this multicenter study, with 137 patients for retrospective training and internal validation, 33 patients for prospective validation, and 37 patients for external validation. All patients received anti-HER2 targeted therapy and underwent pre- and post-treatment CT scans (baseline and at least one follow-up). The proposed deep learning model evaluated the multiple lesions in time series CT images to predict risk probabilities. We further evaluated and validated the risk score of the nomogram combining a two-follow-up lesion-based deep learning model (LDLM-2F), tumor markers, and clinical information for predicting the benefits from treatment (Nomo-LDLM-2F).

**Results:**

In the internal validation and prospective cohorts, the one-year AUCs for Nomo-LDLM-2F using the time series medical images and tumor markers were 0.894 (0.728–1.000) and 0.809 (0.561–1.000), respectively. In the external validation cohort, the one-year AUC of Nomo-LDLM-2F without tumor markers was 0.771 (0.510–1.000). Patients with a low Nomo-LDLM-2F score derived survival benefits from anti-HER2 targeted therapy significantly compared to those with a high Nomo-LDLM-2F score (all *p* < 0.05).

**Conclusion:**

The Nomo-LDLM-2F score derived from multi-lesion and time series CT images holds promise for the effective readout of OS probability in patients with HER2-positive stage IV GC receiving anti-HER2 therapy.

**Critical relevance statement:**

The deep learning model using baseline and early follow-up CT images aims to predict OS in patients with stage IV gastric cancer receiving anti-HER2 targeted therapy. This model highlights the spatiotemporal heterogeneity of stage IV GC, assisting clinicians in the early evaluation of the efficacy of anti-HER2 therapy.

**Key points:**

• Multi-lesion and time series model revealed the spatiotemporal heterogeneity in anti-HER2 therapy.

• The Nomo-LDLM-2F score was a valuable prognostic marker for anti-HER2 therapy.

• CT-based deep learning model incorporating time-series tumor markers improved performance.

**Graphical Abstract:**

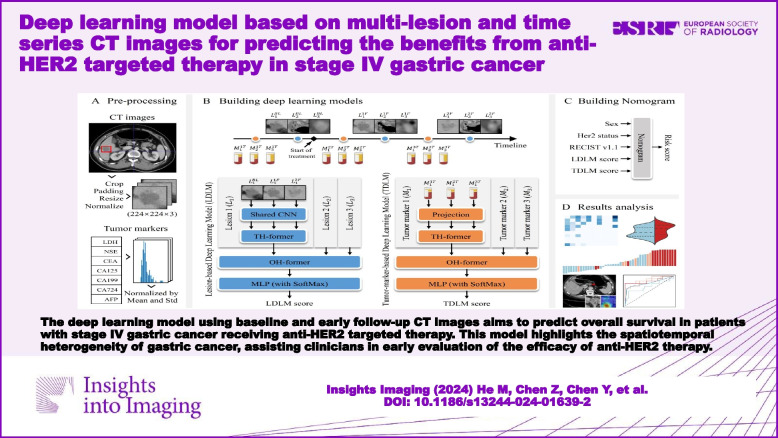

**Supplementary Information:**

The online version contains supplementary material available at 10.1186/s13244-024-01639-2.

## Introduction

Gastric cancer (GC) is globally the fifth most prevalent and third leading cause of cancer-related mortality [1]. Human epidermal growth factor receptor 2 (HER2) overexpression has been detected in 17–30.5% of patients with GC [2, 3]. Anti-HER2 targeted therapy has proven to be a practical treatment approach for GC [4]. In the Trastuzumab for Gastric Cancer (ToGA) study, trastuzumab combined with chemotherapy extended overall survival (OS) to 16 months for patients with HER2-positive advanced GC. However, the ToGA study reported a modest objective response rate of 47.3% [5]. Therefore, identifying the patients who have the potential to benefit from anti-HER2 targeted therapy has long been overdue.

GC is distinguished by high spatiotemporal heterogeneity, which plays a crucial role in resistance to anti-HER2 therapy [6]. The temporal heterogeneity exhibits a change in the HER2 status before and after treatment, while the spatial heterogeneity manifests as discordant HER2 expression between primary and metastatic lesions [7]. The methods to evaluate the spatiotemporal heterogeneity of stage IV GC are lacking in clinical practice. Multi-spot sampling under gastroscopy can only represent a static snapshot of primary tumors at a particular time point, which does not reflect the heterogeneous features of various metastases to targeted therapy over time. Therefore, it is imperative to develop a model that can predict the long-term prognosis of anti-HER2 targeted therapy in both temporal and spatial dimensions so that personalized therapeutics can be managed.

Clinicians mainly use tumor markers and radiographic images to track dynamic tumor changes [8]. RECIST v1.1 is generally used to estimate the therapeutic efficacy in advanced GC, focusing exclusively on the unidimensional measurement of lesions rather than considering the overall landscape. However, the primary tumor cannot be identified as the target lesion due to the hollow nature of the stomach. In contrast, evidence supports the notion that artificial intelligence can reveal longitudinal heterogeneity during treatment [9–12]. The deep learning model developed by Xu et al. utilized baseline and follow-up CT images at months 1, 3, and 6 to predict survival and cancer-specific outcomes for chemoradiation-treated non-small cell lung cancer [8]. Lu et al. used an unlimited number of time series CT images to train the deep learning model and used baseline and two-month follow-up images to predict OS in patients with metastatic colon cancer [9]. No study has used deep learning models based on time series CT images to screen out patients who benefit from anti-HER2 targeted therapy in GC as of yet. We believed that the early radiological changes were worth mining, because they demonstrated the tumor’s responsiveness or resistance to targeted therapy in a temporal dimension.

Multi-lesion and time series images collected in this study could better reflect the spatiotemporal heterogeneity of stage IV GC. We constructed an attention-based deep learning framework that automatically discerns features from multiple lesions of different time points for OS prediction in anti-HER2 targeted therapy. We applied this framework to time series CT images and tumor markers to introduce the lesion-based deep learning model (LDLM) and the tumor marker–based deep learning model (TDLM). Furthermore, we built a nomogram (Nomo-LDLM) by combining the deep learning models with clinical information to achieve accurate early prediction of OS probability.

## Methods

### Data collection

We retrospectively enrolled patients with HER2-positive advanced GC treated with trastuzumab from four centers between November 2011 and November 2019 and prospectively enrolled patients at center 1 between December 2019 and December 2020. The ethics committee of Peking University Cancer Hospital (PUCH) approved this study (No. 2020KT08). OS was defined as the duration from the initiation of anti-HER2 therapy to death from any cause or to the most recent follow-up.

**Fig. 1:**
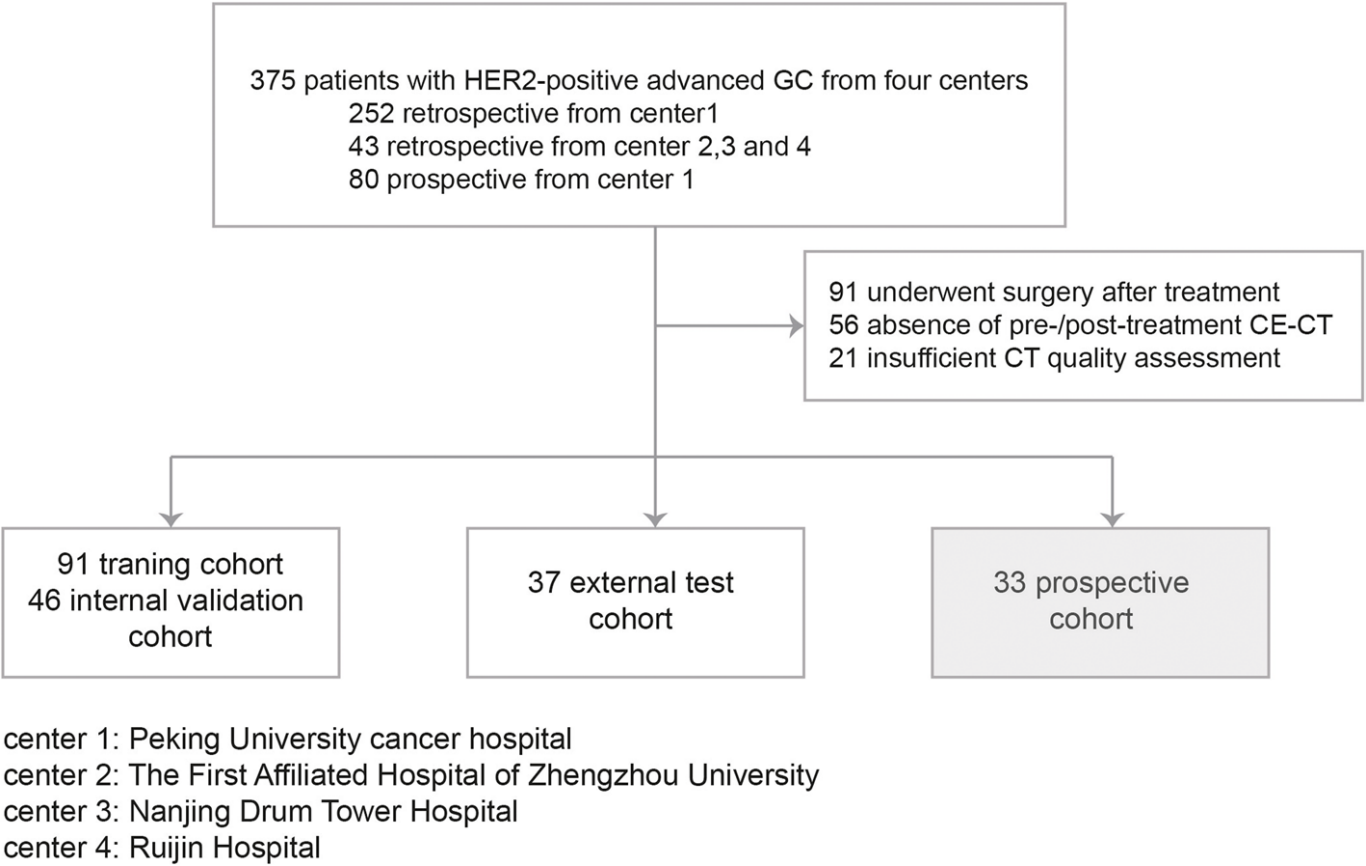
Study participants

The details of patient recruitment are shown in Fig. 1 and Text S1. A total of 375 patients diagnosed as HER2-positive advanced GC were enrolled, of whom 207 patients meeting the criteria were included in the analysis. The retrospectively collected 137 patients from center 1 were randomly split into the training and internal validation cohort in a 2:1 ratio (*n* = 91 and 46, respectively), and the prospectively collected 33 patients were included in the prospective cohort. The external validation cohort included 37 patients from centers 2–4 (25 from The First Affiliated Hospital of Zhengzhou University, 10 from Nanjing Drum Tower Hospital of Nanjing University Medical School, 2 from Ruijin Hospital of Shanghai Jiao Tong University). We included baseline and post-treatment CT images up to four follow-ups in the training cohort for model construction and validated its performance of early prediction in other cohorts only using baseline and post-treatment CT images up to two follow-ups. Moreover, the baseline and post-treatment tumor markers up to two follow-ups were also recorded in center 1 for model improvement (tumor markers in center 2/3/4 were not included). A total of 680 CT scans and 703 times examination of tumor markers were collected. Table S1 displays the protocol details of the CT scans.

### Preprocessing data

The workflow is illustrated in the Graphical abstract. For CT images, two radiologists manually provided bounding boxes at the maximum slice of the primary tumor and target lesions (M.H. and J.Y., both with two years of diagnostic experience) with ITK-SNAP (version 3.8). According to RECIST v1.1, for each patient, the radiologists selected up to five target lesions whose diameters were larger than 10 mm at baseline. Then, the radiologists used bounding boxes to mark each lesion and kept the size of the bounding boxes on the baseline and follow-up images consistent for maintaining scale information. The bounding boxes were then reviewed by a senior radiologist (L.T., with 18 years of diagnostic expertise). All readers were blinded to demographic information.

As shown in the Graphical abstract, the images were processed as follows before being fed into the network: (1) extracting 1.5-fold the annotated bounding box regions from CT slices as the regions of interest (ROIs) of the lesions; (2) symmetrically padding rectangular ROIs to its minimum circumscribed square to generate image patches and then resampling them to 224 × 224 resolution; (3) normalizing the lung metastasis with the window level of -400 and the window width of 1500, and abdominal lesions with the window level of 50 and the window width of 350; and (4) augmenting images by random rotation of -30 to 30° to cope with the uncertainty in clinical settings. We applied the same operations to the upper and lower layers of the annotated lesions and obtained a 3-channel image (224 × 224 × 3) to provide richer contextual information (Fig. S1, S2, Text S2, and Table S2). For tumor markers, we normalized all data with the mean and variance in the training cohort (Table S3).

### Models based on deep learning

We constructed a two-level attention-based deep learning framework using Transformer architecture [13–15]. The first level was the temporal heterogeneity Transformer (TH-former), which modeled the feature changes in lesions or tumor markers over time. The second level, the object heterogeneity Transformer (OH-former), modeled the interactions between the features of multiple lesions or tumor markers and generated a descriptive signature of features for each patient. This framework was instantiated as the LDLM for the lesions and the TDLM for the tumor markers (Fig. S4, Texts S3 and S4). When training LDLM, the CT images put into the model contained a baseline and up to four follow-ups, which improved the robustness of the model by reducing time-dependent signal noise [10, 16]. Then, the CT images obtained at baseline and up to two follow-up visits were incorporated into the model in the validation cohorts for early on-treatment prediction.

For multiple ROIs of a patient, the features were firstly extracted by a feature extractor and then were combined using attention weighting. Figure S5 examples a visualization of feature fusion. We first defined a learnable aggregation vector; then through the attention weighting module of Transformer, the importance of time-serial inter-lesion heterogeneity was passed onto this vector. With this aggregation, each patient would have only one outcome vector, regardless of the number of target lesions. Finally, we used a multi-layer perceptron with softmax layer to generate the predicted OS probability, a continuous variable ranging from 0 to 1, with low values indicating poor OS (< 12 months) and high values indicating good OS (> 12 months). In addition, we used masking operations to deal with the missing data in the clinical settings (Text S5). The code can be found on GitHub https://anonymous.4open.science/r/HER2/.

### Models based on RECIST v1.1 and tumor burden

We established two prognostic models based on RECIST v1.1 and measurements of tumor burden (TB-delta model). For the RECIST model, we assessed the relative changes in diameter between the baseline and the second follow-up based on RECIST criteria. TB-delta was measured on the percentage change of the summed area of target lesions between baseline and the second follow-up. If the patient only had baseline and the first follow-up CT images, the result of RECIST and TB-delta were calculated based on these two examinations.

### Overall model using the nomogram

The deep networks paid much attention to higher-order features [17]. In contrast, the size-based RECIST focused on first-order features, providing a good complement to the deep networks. We constructed a nomogram, called Nomo-LDLM, based on multiple variables (LDLM score, TDLM score, RECIST, and clinical information) through Cox regression to achieve complementary advantages. We also constructed a nomogram without the TDLM score, Nomo-w/o TDLM, for performance comparison in the external validation cohort without tumor markers.

### Explanation of deep learning models

The core module of the Transformer blocks (TH-former and OH-former) modeled complex relationships between multiple components (time points and objects). We depicted the last layer of the Transformer as attention maps, which highlighted the contribution of different time points and distinguished the relationship between lesions from different organs in high- and low-risk groups. In particular, we used Gradient-weighted Class Activation Mapping (GradCAM) algorithm to generate the heatmap visualization of the LDLM. The high-response regions in the heatmap represented that the model paid more attention to the part of lesions, indicating a strong relationship between the lesions and risk probability at the pixel level [18].

### Statistical analysis

Data were described as means with standard deviation. The Kruskal–Wallis test was used for quantitative variables, and the Wilcoxon signed-rank test was used for correlated samples. Pearson’s chi-square and Fisher’s test were used for qualitative variables. Concordance index (C-index) and one-year AUC were used to compare the performance of the models in predicting OS. The Youden index was used to select the best cutoff value in the training cohort and stratified patients into the low- or high-risk group in other cohorts. Kaplan–Meier analysis and log-rank test were performed for OS comparison in the two groups. The calibration curve and Hosmer–Lemeshow test were performed to analyze the predictive abilities of the nomogram. Statistical analyses were conducted using R 4.1.3 and Python 3.7. A *p* < 0.05 indicated a statistically significant difference.

## Results

### Clinical characteristics

This study finally included 207 patients with HER2-positive stage IV GC from 4 centers (Table 1). Kaplan–Meier analysis in different centers and cohorts revealed no significant difference in survival (*p* = 0.22 and 0.064, respectively, Fig. S6). A total of 680 CT scans were used in the study, and 765 lesions (207 primary and 558 target lesions, average 3.70 ± 1.23 per patient) involving 10 anatomical sites were examined. Only 17 individuals, accounting for 8% of the total, underwent just one follow-up CT examination, and other patients collected baseline and at least two follow-up CT images. A total of 4104 bounding boxes were delineated on the arterial and venous phases of the full-body CT (only the venous phase for liver metastases). No significant differences in the number and area of target lesions were found among the four cohorts (*p* > 0.0083, Bonferroni correction, Tables S1 and S4).

**Table 1:** Characteristics of patients, lesions, and tumor markers

	**Training cohort (*n* = 91)**	**Internal validation cohort (*n* = 46)**		**External validation cohort (*n* = 37)**	**Prospective cohort (*n* = 33)**	***p* value**
**Patients**						
Age (mean ± SD)	60.8 ± 10.7	60.0 ± 12.8		63.8 ± 10.5	67.0 ± 19.5	0.217^#^
Sex (*n* (%))						
Male	70 (76.92)	37 (80.43)		31 (83.78)	25 (75.76)	0.802^*^
Female	21 (23.08)	9 (19.57)		6 (16.22)	8 (24.24)	
HER2 expression (*n* (%))						
HER2 2 + /FISH +	21 (23.08)	8 (17.39)		14 (37.84)	8 (24.24)	0.180^*^
HER2 3 +	70 (76.92)	38 (82.61)		23 (62.16)	24 (72.73)	
No. line of therapy (*n* (%))						
1	76	36		31	23	0.319^*^
2	13	9		5	6	
3 or more	2	1		1	4	
**Lesions**						
Total number	331	161		146	127	
Anatomic position (*n* (%))						
Stomach	91 (27.49)	46 (28.57)	37 (25.34)		33 (25.98)	0.930^*^
Lymph node	120 (36.25)	57 (35.40)	50 (34.25)		40 (31.50)	
Liver	91 (27.49)	45 (27.95)	50 (34.25)		40 (31.50)	
Adrenal gland	9 (2.72)	3 (1.86)	3 (2.05)		2 (1.57)	
Spleen	1 (0.30)				1 (0.79)	
Peritoneum	7 (2.11)	4 (2.48)	3 (2.05)		4 (3.15)	
Soft tissue	2 (0.60)	1 (0.62)				
Bone	2 (0.60)					
Lung	8 (2.42)	4 (2.48)	3 (2.05)		5 (3.94)	
Other		1 (0.62)			2 (1.57)	
**Bounding boxes**						
Total number (*n* (%))	2125	728	682		569	
Baseline (BL)	561 (26.40)	271 (37.23)	239 (35.04)		207 (36.38)	
First follow-up (1F)	559 (26.31)	265 (36.40)	232 (34.02)		203 (35.68)	
Second follow-up (2F)	462 (21.74)	192 (26.37)	211 (30.94)		159 (27.94)	
Third follow-up (3F)	333 (15.67)					
Fourth follow-up (4F)	210 (9.88)					

*FISH* Fluorescence in situ hybridization

^#^ANOVA test

^*^Chi’s square test or Fisher’s test

### Development and validation of LDLMs

The model was trained with 2125 bounding boxes of 331 lesions in the training cohort and then predicted the risk probability of the patients longitudinally over time. We found that the deep learning model based on two follow-ups was overall better than LDLM-BS and LDLM-1F (baseline (BS); one follow-up (1F); two follow-ups (2F), Table 3), with the one-year AUC of internal validation, external validation, and prospective cohorts 0.844 (0.673–0.971), 0.683 (0.350–0.957), and 0.690 (0.419–0.962), respectively.

**Fig. 2:**
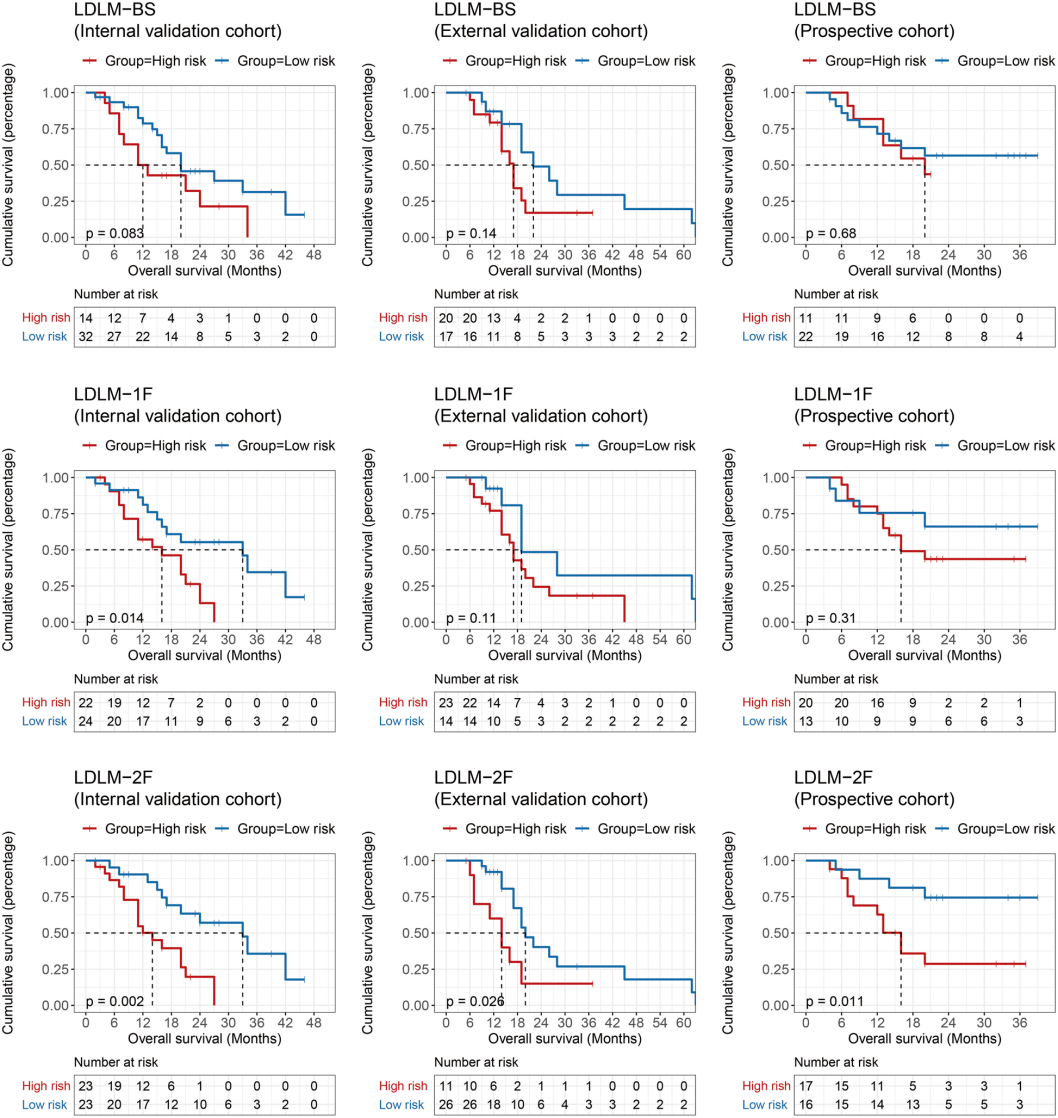
Overall survival Kaplan–Meier analysis was performed with the cutoff value derived from the training cohort by the Youden index. Internal validation, external validation, and prospective cohorts were stratified into high- and low-risk groups by LDLM with no follow-up to two follow-ups (*p* < 0.05, log-rank test)

We selected the optimal cutoff value according to the Youden index on the training cohort. Patients with predicted risk probability greater than the cutoff value were classified as the high-risk group, and those with lower risk probability were classified as the low-risk group. Kaplan–Meier analysis was performed between the two groups to compare the prognostic stratification ability of models based on the baseline with no follow-up or up to two follow-up CT scans (Fig. 2). We found that the LDLM-BS did not yield a statistical disparity in prognosis between high- and low-risk groups among the three cohorts (*p* > 0.05). With the addition of the follow-up CT scans, the stratification in OS between the high- and the low-risk groups gradually became more separable (LDLM-2F: all *p* < 0.05, log-rank test).

### Comparison between LDLM-2F and other models

**Fig. 3:**
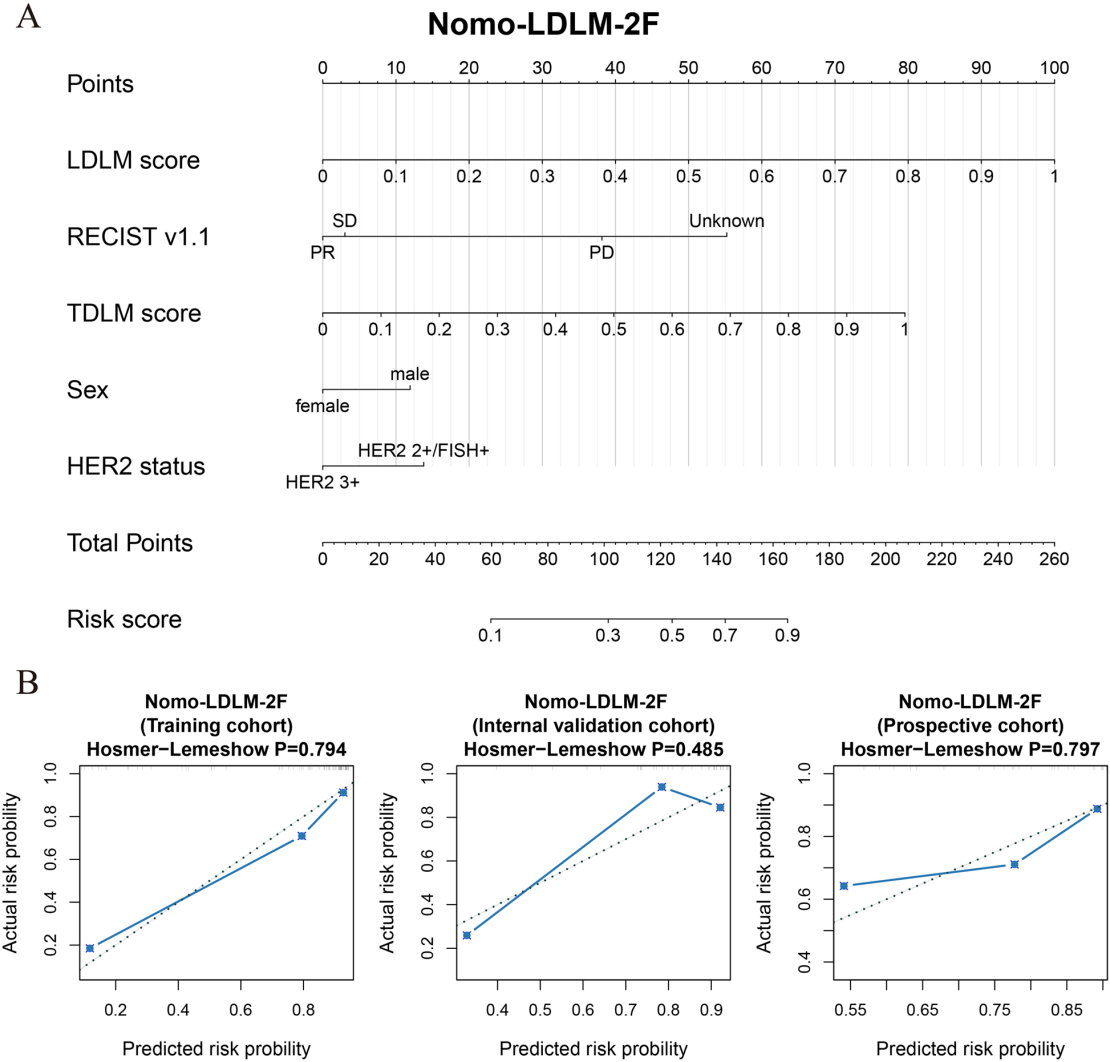
Developed deep learning nomogram. The nomogram was built in the training cohort, incorporating the deep learning signature, RECIST, tumor markers, sex, and HER2 status (**A**). Calibration curve of the nomogram in the training, internal validation, and prospective cohorts (**B**)

**Fig. 4:**
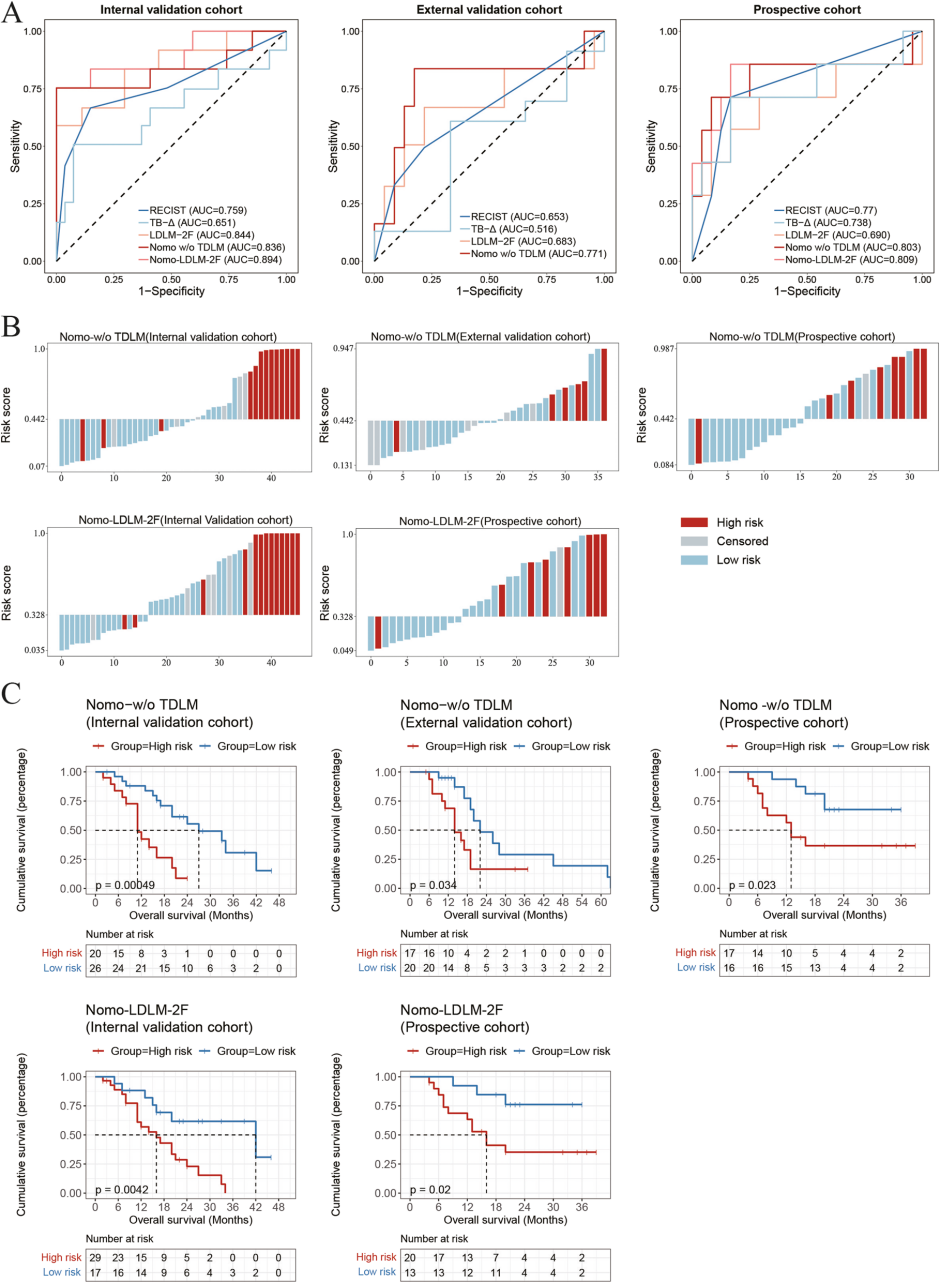
**A** The ROC curves and areas under ROC (AUCs) of RECIST, TB-delta, LDLM-2F, and Nomo-LDLM-2F to predict one-year survival in internal validation, external validation, and prospective cohort. **B**, **C** Distribution of the LDLM score and its corresponding prognostic risk group in the internal validation, external validation, and prospective cohorts

As shown in Table 2, we established eight prognostic models of RECIST, TB-delta, LDLM-BS, LDLM-1F, LDLM-2F, TDLM, Nomo-LDLM-2F, and Nomo-w/o TDLM (Fig. S7). Figure 3 shows the Nomo-LDLM-2F and its one-year survival calibration curve. The predicted results of Nomo-LDLM-2F were in good agreement with the actual results (AUC of training = 0.891 (0.785–0.967), internal validation = 0.894 (0.728–1.000), and prospective = 0.809 (0.561–1.000)). In the external validation cohort, the Nomo-w/o TDLM also reached an AUC of 0.771 (0.510–1.000) (Fig. 4A).

**Table 2:** Descriptions for main models

Models	CT images			Tumor markers	RECIST	Clinical information	Descriptions
	**BS**	**1F**	**2F**				
RECIST	√	√	√				Based on RECIST 1.1, treatment response is defined as PR, SD, and PD
TB-Δ	√	√	√				Change in percentage in tumor size
LDLM-BS	√						LDLM based on baseline CT scans
LDLM-1F	√	√					LDLM based on baseline and the first one follow-up CT scans
LDLM-2F	√	√	√				LDLM based on baseline and the first two follow-up CT scans
TDLM				√			Based on serial tumor markers
Nomo-w/o TDLM	√	√	√		√	√	Nomogram integrated by LDLM, RECIST, and clinical information
Nomo-LDLM-2F	√	√	√	√	√	√	Nomogram integrated by LDLM, TDLM, RECIST, and clinical information

*BS* Baseline, *1F* One follow-up, *2F* Two follow-ups, *LDLM-2F* Two-follow-up lesion-based deep learning model, *Nomo-LDLM-2F* Nomogram based on LDLM-2F, *Nomo-w/o TDLM* Nomogram without TDLM, *TB* Tumor burden, *TDLM* Tumor marker–based deep learning model

In terms of prognostic prediction, the RECIST performed relatively well; however, it failed to stratify risk in all cohorts (Fig. S8). The Nomo-w/o TDLM or Nomo-LDLM-2F outperformed the other models in the four cohorts, suggesting that the addition of follow-up images provided additional information on temporal heterogeneity, enabling more precise prognostic stratification. The nomogram with TDLM performed the best in the internal validation and prospective cohorts, indicating that the addition of tumor markers refined the multidimensional model (Table 3, Fig. 4B, C).

**Table 3:** Performance comparisons of different models in predicting overall survival and AUCs in predicting one-year survival in four cohorts

Models	C-index (95% CI)	HR (*p* value)	AUC (95% CI)
Training cohort (*n* = 91)			
RECIST	0.648 (0.572–0.717)	< 0.0001	0.753 (0.628–0.860)
TB-Δ	0.613 (0.482–0.696)	0.0447	0.675 (0.514–0.788)
LDLM-2F	0.775 (0.712–0.829)	< 0.0001	0.879 (0.775–0.961)
TDLM	0.717 (0.641–0.793)	< 0.0001	0.780 (0.658–0.885)
Nomo-LDLM-2F	**0.807** (0.743–0.865)	< 0.0001	**0.891** (0.785–0.967)
Internal validation cohort (*n* = 46)			
RECIST	0.652 (0.523–0.771)	0.0133	0.759 (0.572–0.937)
TB-Δ	0.594 (0.483–0.724)	0.0456	0.651 (0.437–0.862)
LDLM-2F	0.725 (0.601–0.836)	0.0002	0.844 (0.673–0.971)
TDLM	0.718 (0.589–0.829)	0.0029	0.867 (0.739–0.961)
Nomo-LDLM-2F	**0.752** (0.635–0.871)	< 0.0001	**0.894** (0.728–1.000)
External validation cohort (*n* = 37)			
RECIST	0.627 (0.516–0.753)	0.0166	0.653 (0.396–0.930)
TB-Δ	0.527 (0.407–0.788)	0.7578	0.516 (0.342–0.896)
LDLM-2F	0.669 (0.503–0.836)	0.0865	0.683 (0.350–0.957)
Nomo-w/o TDLM	**0.709** (0.562–0.855)	0.0106	**0.771** (0.510–1.000)
Prospective cohort (*n* = 33)			
RECIST	0.644 (0.440–0.783)	0.1710	0.770 (0.547–0.959)
TB-Δ	0.630 (0.446–0.768)	0.0226	0.738 (0.427–0.965)
LDLM-2F	0.726 (0.566–0.877)	0.0052	0.690 (0.419–0.962)
TDLM	0.595 (0.475–0.743)	0.3411	0.678 (0.440–0.915)
Nomo-LDLM-2F	**0.741** (0.570–0.882)	0.0095	**0.809** (0.561–1.000)

*AUC* Area under the curve, *C-index* Concordance index, *HR* Hazard ratio

### GradCAM for visualization of regions highlighted in LDLM

**Fig. 5:**
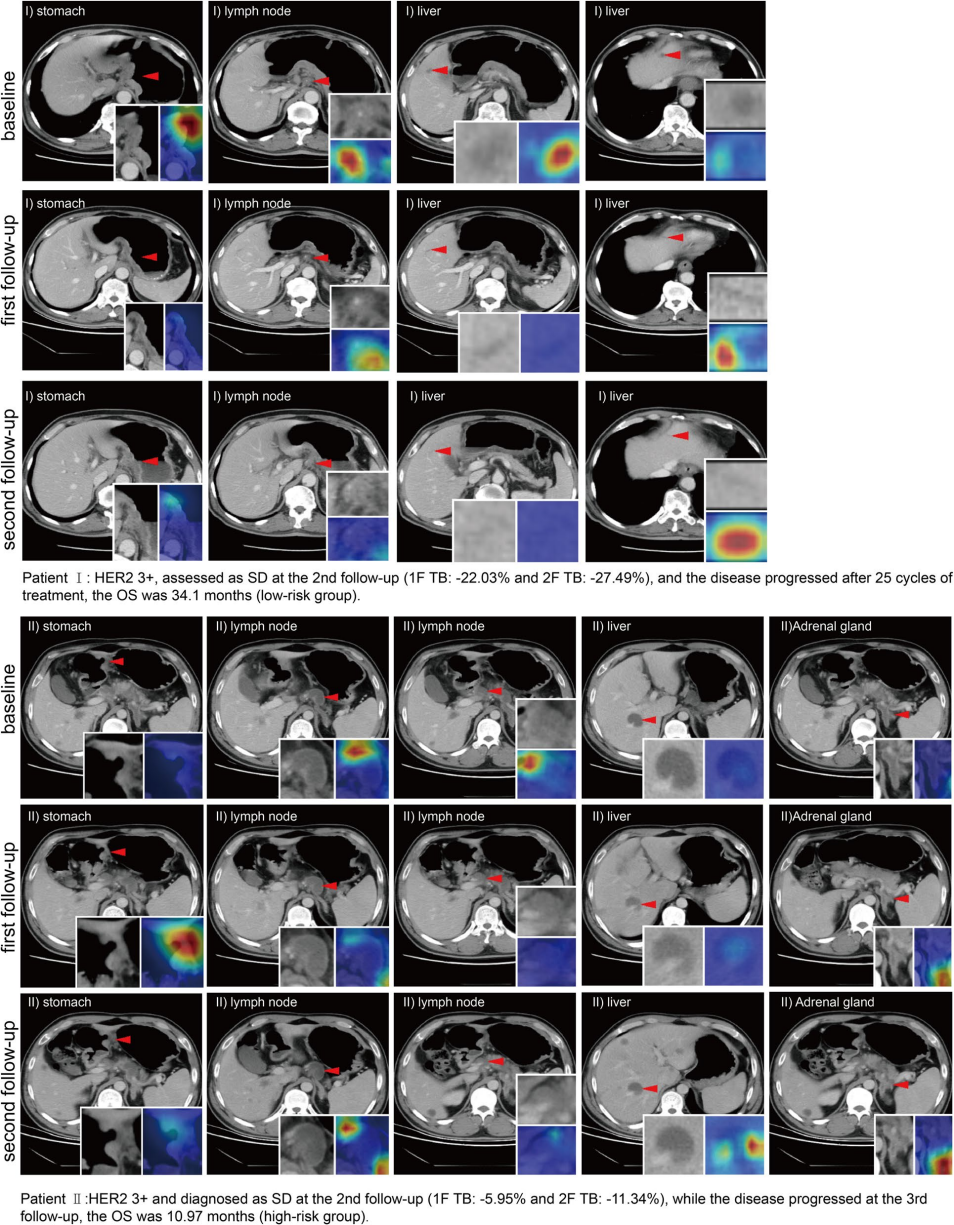
Visualization of the LDLM model using the GradCAM method. Attention maps for two patients with stage IV GC are shown in this figure. They were both assessed as SD at the second follow-up and had varying survival times. The LDLM model paid more attention to the strong response areas valuable for OS prediction. LDLM, lesion-based deep learning model

**Fig. 6:**
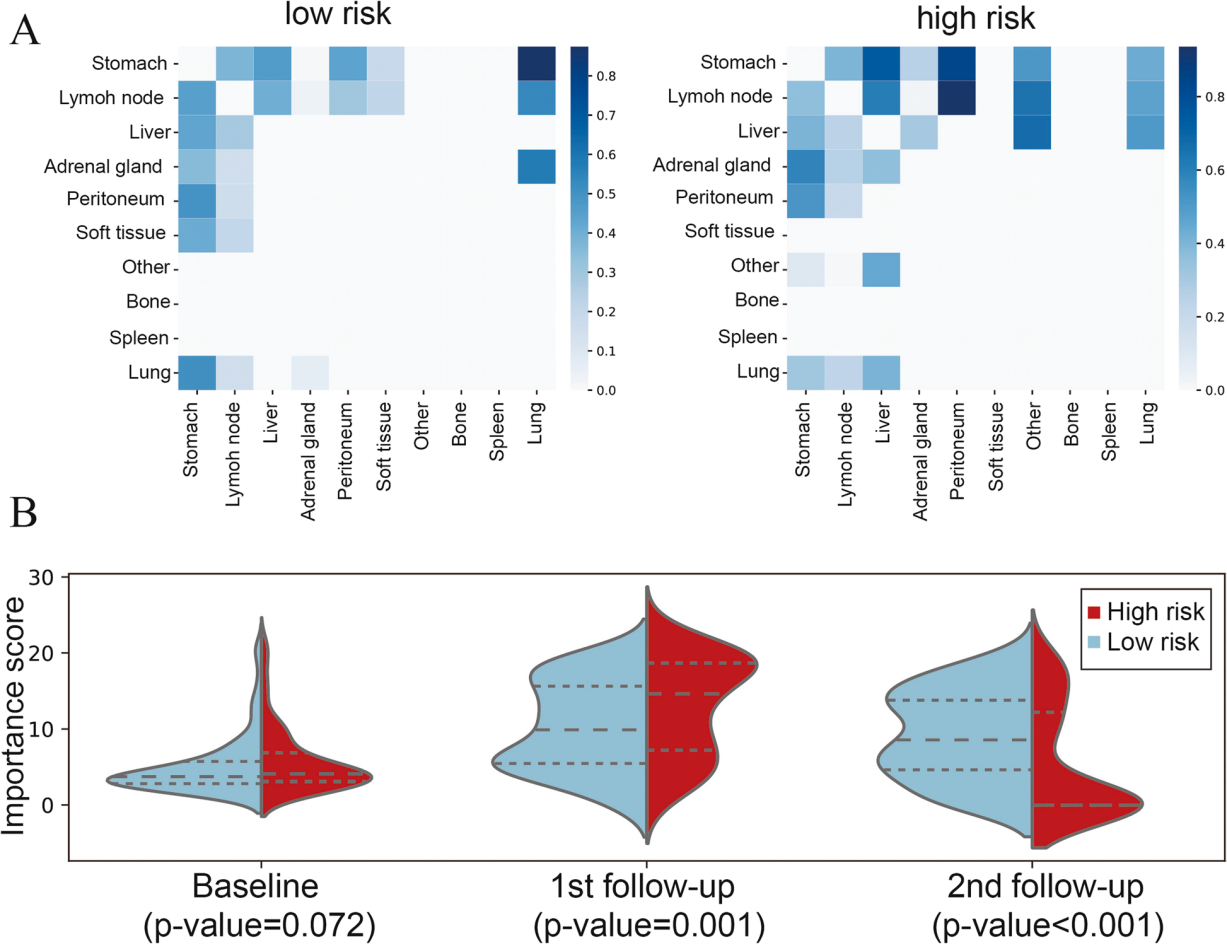
The importance map analyzed on the attention patterns of the relationship between target lesions of different organs (**A**). The importance of attention patterns of different time points differed in the high- and low-risk groups (**B**)

We used GradCAM to localize the saliency information correlated to prognosis for clustering lesions where tumors or peripheral areas were activated. GradCAM identified the superiority of LDLM in revealing the spatiotemporal differences between high- and low-risk groups. Figure 5 displays two patients, one from the low-risk group (I) HER2 3 + , assessed as SD at the second follow-up (TB = -27.49%); the disease progressed after 25 cycles of treatment, and the OS was 34.1 months. GradCAM mapping focused on the primary tumor and the marginal part of the lymph node. Another patient in the high-risk group (II) was also HER2 3 + and diagnosed as SD at the second follow-up (TB = -11.34%); the disease progressed at the third follow-up, and the OS was 10.97 months. In the GradCAM maps, LDLM paid great attention to the primary tumor and liver metastases. As shown in Fig. 6A, the lymph nodes had a strong synergistic correlation with peritoneal metastases in the high-risk group. In general, the lymph nodes had a high correlation with other metastases. We also observed the importance of attention patterns on different time points in the high- and low-risk groups (Fig. 6B). LDLM focused more on the first follow-up in the high-risk group compared with the low-risk group, suggesting that patients with poor prognosis were more likely to occur drug resistance at an early stage.

## Discussion

We established a nomogram built with clinical data and radiological signatures using multi-lesion and time series CT images from multiple centers, which could estimate the OS outcomes of patients with stage IV GC treated with anti-HER2 targeted therapy at an early stage.

The heterogeneity between primary and metastatic lesions is crucial for treatment evaluation and clinical decision-making. The high lesion-level heterogeneity of GC makes it difficult to accurately assess the overall treatment response from a single lesion [19, 20]. Most previous radiomics studies used only primary [9, 21] or target lesions [10] to predict prognosis. In our ablation experiments, we found that using primary lesions alone was not effective while using target lesions alone achieved higher but unsatisfactory performance. Our model simultaneously considered primary and target lesions, achieving promising performance and suggesting that the complementary information was useful (Table S5). We exploited GradCAM maps to visualize the importance of inter-tumor interactions in LDLM. The lesion interaction pattern of high-risk patients was more complex. It focused the most on the relationship between peritoneal metastases with primary tumors and lymph nodes, which might be related to the highly invasive biological behavior.

Furthermore, tracking tumor evolution is essential to predict the prognosis of targeted therapies. Previous radiomics studies focused on pre-treatment images at a single time point. In this study, we provided explicit temporal information by introducing temporal position, enabling the model to distinguish baseline from follow-up scans. Embedding without temporal position would weaken the model’s performance because it focused only on the overall characteristics of a patient at multiple time points rather than sequential information (Table S6). The follow-up CT scans are a routine part of antitumor treatment practice. Our model provided additional dynamic information over time without extra invasive examinations, helping clinicians assess patients’ suitability for anti-HER2 targeted therapy.

Further, previous studies used only the cross-entropy loss function [22, 23] or survival loss [24, 25] for training the prognostic model. However, Table S7 shows that using “${l}_{ce}+{l}_{surv}$” outperformed using either alone. The cross-entropy loss paid more attention to the discernible representations between different groups, and the survival loss was responsible for ordering the relationship between all samples, providing additional information for marginal samples. We verified through ablation experiments that aggregating both losses improved the model’s performance (Text S6 and Tables S7 and S8).

This study had several limitations. We included patients from multiple centers; however, the sample size was still limited. Hence, we collected up to four follow-up CT images in the training cohort to reduce time-dependent signal noise. We also set multiple random seeds to ensure reproducibility (Text S7 and Table S9). Furthermore, although we used the bounding boxes to reduce workload, the radiologists still manually segmented the ROIs. Moreover, this was a single imaging modality prediction model. We hope to incorporate other modalities such as magnetic resonance imaging or pathological images to make it more reliable and robust. We have developed in-house software at our center (Additional file 2: video), experienced fellows quickly annotated the ROIs for risk probability calculations, and combine clinical information and RECIST evaluation results to assess patients’ long-term prognosis. Thus, data from larger patient populations, preferably more comprehensive data collected prospectively, containing multimodal information and comorbid factors, are required for validating its generalization and accuracy.

In conclusion, this study demonstrated, based on baseline and early follow-up CT images, that deep learning models could predict the OS in patients with stage IV GC receiving anti-HER2 targeted therapy. The analysis of multi-lesion and time series CT images can simultaneously focus on the spatiotemporal heterogeneity of stage IV GC, which may help clinicians make early treatment decisions.

### Supplementary Information

**Additional file 1: Text S1.** Details of patient recruitment. **Text S2.** Details of the preprocessing procedure. **Text S3.** Details of LDLM. **Text S4.** Details of TDLM. **Text S5.** The details of missing data processing. **Text S6.** **Text S7.** **Table S1.** CT protocol of the four centers. **Table S2.** Normalization parameters for different centers. **Table S3.** Normalization parameters for different tumor markers. **Table S4.** Statistics of annotation information. **Table S5.** Ablation study for lesions input to the model. **Table S6.** Ablation study for temporal position embedding on the validation cohort. **Table S7.** Ablation study for different loss functions on the validation cohort. **Table S8.** Impact of mini-batch size for survival loss on the validation cohort. **Table S9.** Reproducibility analysis of different random seeds on the validation cohort. **Table S10.** Performance comparisons of all models in predicting overall survival and AUCs in predicting one year survival in four cohorts. **Figure S1.** A sample for the preprocessing of CT images. **Figure S2.** A typical example for input, including bounding boxes at baseline and at the first two follow-up visits. **Figure S3.** Architecture of CNN-based feature extractor based on ResNet-18. **Figure S4.** Details of modules. **Figure S5.** Through an attention-weighting mechanism, Time-heterogeneity Transformer and Object-heterogeneity Transformer combined different lesion features from a patient at different time points to generate Lesion-based Deep Learning scores. **Figure S6.** Overall survival analysis for different centers and cohorts. **Figure S7.** Developed nomogram without TDLM (Nomo-w/o TDLM). **Figure S8.** Overall survival Kaplan–Meier analysis was performed in the training, internal validation, external test, and prospective cohorts stratified by RECIST 1.1 (*p* < 0.05, log-rank test). **Figure S9.** A sample case used to illustrate how the masking operation coped with the absence of certain tumor markers.

**Additional file 2:** Supplementary video.

## Data Availability

The datasets generated or analyzed during the study are not publicly available but are available from the corresponding author on reasonable request. The code in this study can be found on GitHub https://anonymous.4open.science/r/HER2/.

## Abbreviations

1F: One follow-up
2F: Two follow-ups
BS: Baseline
C-index: Concordance index
GC: Gastric cancer
GradCAM: Gradient weighted Class Activation Mapping
HER2: Human epidermal growth factor receptor 2
LDLM: Lesion-based deep learning model
OS: Overall survival
RECIST: Response Evaluation Criteria in Solid Tumors
ROI: Region of interest
TB: Tumor burden
TDLM: Tumor marker–based deep learning model
ToGA: Trastuzumab for Gastric Cancer
