# Supplementary material for: Deep learning model based on multi-lesion and time series CT images for predicting the benefits from anti-HER2 targeted therapy in stage IV gastric cancer
Source: Insights Imaging. 2024 Feb 27;15:59. doi: 10.1186/s13244-024-01639-2 (PMC10899559; doi:10.1186/s13244-024-01639-2)
Supplement: Supplementary file 1 — Additional file 1: Text S1. Details of patient recruitment. Text S2. Details of the preprocessing procedure. Text S3. Details of LDLM. Text S4. Details of TDLM. Text S5. The details of missing data processing. Text S6. Text S7. Table S1. CT protocol of the four centers. Table S2. Normalization parameters for different centers. Table S3. Normalization parameters for different tumor markers. Table S4. Statistics of annotation information. Table S5. Ablation study for lesions input to the model. Table S6. Ablation study for temporal position embedding on the validation cohort. Table S7. Ablation study for different loss functions on the validation cohort. Table S8. Impact of mini-batch size for survival loss on the validation cohort. Table S9. Reproducibility analysis of different random seeds on the validation cohort. Table S10. Performance comparisons of all models in predicting overall survival and AUCs in predicting one year survival in four cohorts. Figure S1. A sample for the preprocessing of CT images. Figure S2. A typical example for input, including bounding boxes at baseline and at the first two follow-up visits. Figure S3. Architecture of CNN-based feature extractor based on ResNet-18. Figure S4. Details of modules. Figure S5. Through an attention-weighting mechanism, Time-heterogeneity Transformer and Object-heterogeneity Transformer combined different lesion features from a patient at different time points to generate Lesion-based Deep Learning scores. Figure S6. Overall survival analysis for different centers and cohorts. Figure S7. Developed nomogram without TDLM (Nomo-w/o TDLM). Figure S8. Overall survival Kaplan–Meier analysis was performed in the training, internal validation, external test, and prospective cohorts stratified by RECIST 1.1 (p < 0.05, log-rank test). Figure S9. A sample case used to illustrate how the masking operation coped with the absence of certain tumor markers. [file 13244_2024_1639_MOESM1_ESM.pdf]

Deep learning model based on multi-lesion and time series CT images for predicting the benefits from anti-HER2 targeted therapy in stage IV gastric cancer

ELECTRONIC SUPPLEMENTARY MATERIAL

Text S1. Details of patient recruitment.

This retrospective and prospective study was a multi-center study with 170 patients from center 1 (Peking University Cancer Hospital & Institute in China, 33 patients were prospectively enrolled), 25 patients from center 2 (The First Affiliated Hospital of Zhengzhou University in China), 10 patients from center 3 (Nanjing Drum Tower Hospital of Nanjing University in China) and 2 patients from center 4 (Ruijin Hospital of Shanghai Jiao Tong University in China).
Patients were divided into four cohorts: training cohort, internal validation cohort, external validation cohort and prospective cohort. The training and internal validation cohort contained 137 patients treated between November 2011 and November 2019 in center 1. The external validation cohort contained 37 patients between July 2016 and November 2019 in center 2,3 and 4. The prospective cohort included 33 patients between December 2019 and December 2020 from center 1.

The inclusion and exclusion criteria were shown as follows.
The inclusion criteria were: (1) stage IV GC confirmed by gastroscopic biopsy and CT imaging, without a chance for surgery; (2) histopathologically proved HER2 positive by immunohistochemical staining 3+ or 2 +/fluorescence in situ hybridization +; (3) chest–abdomen–pelvis contrast-enhanced CT examinations at baseline (within 1 month before treatment initiation) and at least the first follow-up scans (8–12 weeks); and (4) existed measurable target lesions or evaluable lesions according to RECIST.
The exclusion criteria were: (1) absence of pre-/post- treatment contrast-enhanced CT; (2) insufficient CT quality; (3) combined with other malignant tumors.
The workflow of study population is shown in Fig. 1.

Text S2. Details of the preprocessing procedure.

The data from different centers had different distributions due to different equipment and imaging parameters (Table S1). For example, computed tomography (CT) images from center 2 were naturally brighter than those from other centers because the arterial phase interval time in center 2 was 10 s earlier than in other centers, which caused a domain gap and degraded the model performance [1]. We simply used the separation normalization for different centers instead of adversarial training or self-training to alleviate the domain gap because we did not include data from other centers during training in this study. The parameters of normalization distinguished by different centers are shown in Table S2.

Text S3. Details of LDLM.

In this study, we used the Lesion-based Deep Learning Model (LDLM) to predict the risk scores based on CT images. The regions of interest (ROIs) annotated by radiologists were preprocessed and passed through a convolutional neural network (CNN) for extracting features. We used the popular CNN, ResNet [2], as the feature extractor. ResNet has five different versions, including ResNet-18, ResNet-34, ResNet-50, ResNet-101, and ResNet-152. The larger the number X (ResNet-X), the deeper the model and the greater the number of parameters, always implying that it is easier to overfit with a small amount of data. In this study, we had only 91 patients (with 331 lesions) for training. Thus, we chose ResNet-18 for extracting features from ROIs to make a tradeoff between the efficacy of clinical application and the overfitting problem. As shown in Figure S3, ResNet-18 had 17 convolutional layers, each followed by a batch normalization layer and an activation function (using ReLU in standard implementation) and a fully connected layer. Except for the first convolutional layer, the remaining convolutional layers formed a "Basic Block" for every two layers, and each "Basic Block" used a residual path to enhance the flow of information while preventing the gradient from disappearing during training [2]. To build a CNN-based feature extractor, we removed the last fully connected layer and used the output from the last convolutional layer (pooled by "Adaptive Avg Pool") as the feature of each image. The implementation details of ResNet-18 can be found in the PyTorch official documentation[1].
We supposed that the images at T time points were collected, and the radiologist found N lesions at each time point in the images. The images of the nth lesion at the tth time point were indicated by $\mathbf{X}_{\mathbf{n}}^{\boldsymbol{t}} \in \mathbb{R}^{H\times W\times C}$. The image sequence of the nth lesion at T time points was defined as $\mathbf{X}_n = \{\mathbf{X}_n^t\} \in \mathbb{R}^{T\times H\times W\times C}, t = 1,2,\ldots,T$. Moreover, $\mathbf{E}_{\mathbf{n}} = \{E_n^t\} \in \mathbb{R}^T$ indicated the number of days by which T time points were offset from baseline. Based on annotations provided by radiologists, ROIs $\widehat{\mathbf{X}}_n = \{\widehat{\mathbf{X}}_n^t\} \in \mathbb{R}^{T\times H'\times W'\times C}$ were obtained after cropping, scaling, padding, and normalization. ResNet-18 ($\mathcal{E}$) mapped $\widehat{\mathbf{X}}_n$ into features $\mathbf{F}_n = \{\mathbf{F}_n^t\} = \{\mathcal{E}(\widehat{\mathbf{X}}_n^t)\} \in \mathbb{R}^{T\times D}$. The features of the nth lesion went through the Time-Heterogeneity Transformer (TH-former) to model the temporal characteristics of the lesion at different time points, expressed as $\mathbf{I}_n = \mathrm{TH-former}(\mathbf{F}_n, \mathbf{E}_n) \in \mathbb{R}^D$. Fig. S4B illustrates the TH-former's architecture.
Before introducing the details of the TH-former, we first described the Multi-Head Attention (MHA) module [6] (as shown in Figure S4A), which was the core module of the TH-former and OH-former. We supposed that the input of an MHA was $\mathbf{F}_{in} \in \mathbb{R}^{M\times D}$, where M is the number of objects. Further, $D' = D/H$ dimensions were assigned for each head, with H heads. The feature in the hth head was noted as $\mathbf{F}_{in}^{(h)} \in \mathbb{R}^{M\times D'}$. The calculation process of MHA was described as

$$\mathbf{F}_{out}^{(h)} = \mathrm{MHA}\left(\mathbf{F}_{in}^{(h)}\right) = \mathrm{Scaled}\left(\alpha\left(\mathbf{F}_{in}^{(h)}\right)\beta\left(\mathbf{F}_{in}^{(h)}\right)^T\right)\gamma\left(\mathbf{F}_{in}^{(h)}\right) \in \mathbb{R}^{M\times D'}, (S1)$$

$$\mathbf{F}_{out} = \mathbf{F}_{out}^{(1)} \oplus \mathbf{F}_{out}^{(2)} \oplus \cdots \oplus \mathbf{F}_{out}^{(H)} \in \mathbb{R}^{M\times D},$$

where $\alpha(\cdot), \beta(\cdot)$, and $\gamma(\cdot)$ are fully connected layers. $\mathrm{Scaled}\left(\alpha\left(\mathbf{F}_{in}^{(h)}\right)\beta\left(\mathbf{F}_{in}^{(h)}\right)^T\right)$ is considered as the attention map $\mathbf{A}$ in MHA describes the connection between each two objects. $\oplus$ indicates connect operation at the channel level.
The TH-former accepted two inputs: (1) features of ROIs at each time point $\mathbf{F}_n$; and (2) the offset days of each time point $\mathbf{E}_n$. Inspired by ViT[7], we initialized a learnable parameter $\mathbf{F}_n^0$, as a distillation token to aggregate information from different time points. For offset days, we applied fully connected layer φ to map them into the embedding space with D dimensions, defined as $\mathbf{L}_n = \{\mathbf{L}_n^t\} \in \mathbb{R}^{T\times D}, \mathbf{L}_n^t = \varphi(E_n^t), \varphi: \mathbb{R}^1 \longrightarrow \mathbb{R}^D$. Specifically,

$$\mathbf{L}_n^{t,(i)} = \varphi(E_n^t)^{(i)} := \begin{cases} \sin\left(\dfrac{1}{10000^{\frac{2k}{d}}} \cdot E_n^t\right), \text{if } i = 2k \\ cos\left(\dfrac{1}{10000^{\frac{2k}{d}}} \cdot E_n^t\right), \text{if } i = 2k+1 \end{cases},$$

where (i) represents the ith component that generates the D-dimensional feature. Similar to $\widehat{\mathbf{F}}_n$, we added learnable

[1] https://pytorch.org/vision/stable/_modules/torchvision/models/resnet.html

parameters as the distillation token. Therefore, the inputs of the TH-former became $\hat{\mathbf{F}}_n = \{\mathbf{F}_n^0, \mathbf{F}_n^t\} \in \mathbb{R}^{(T+1)\times D}$ and $\hat{\mathbf{L}}_n = \{\mathbf{L}_n^0, \mathbf{L}_n^t\} \in \mathbb{R}^{(T+1)\times D}$. The detailed calculation process of the TH-former was

$$\mathbf{I}_n^{\mathrm{MHA}_1} = (\hat{\mathbf{F}}_n + \hat{\mathbf{L}}_n) + \mathrm{Norm}\left(\mathrm{MHA}(\hat{\mathbf{F}}_n + \hat{\mathbf{L}}_n)\right),$$

$$\mathbf{I}_n^{\mathrm{FC}_1} = \mathbf{I}_n^{\mathrm{MHA}_1} + \mathrm{Norm}\left(\mathbf{W}_{\mathrm{MHA}_1}^{\mathrm{T}} \mathbf{I}_n^{\mathrm{MHA}_1} + \mathbf{b}_{\mathrm{MHA}_1}\right),$$

$$\mathbf{I}_n^{\mathrm{MHA}_2} = (\mathbf{I}_n^{\mathrm{FC}_1} + \hat{\mathbf{L}}_n) + \mathrm{Norm}\left(\mathrm{MHA}(\mathbf{I}_n^{\mathrm{FC}_1} + \hat{\mathbf{L}}_n)\right),$$

$$\mathbf{I}_n^{\mathrm{FC}_2} = \mathbf{I}_n^{\mathrm{MHA}_2} + \mathrm{Norm}\left(\mathbf{W}_{\mathrm{MHA}_2}^{\mathrm{T}} \mathbf{I}_n^{\mathrm{MHA}_2} + \mathbf{b}_{\mathrm{MHA}_2}\right),$$

$$\mathbf{I}_n = \mathrm{Distillation}(\mathbf{I}_n^{\mathrm{FC}_2}),$$

where the "Distillation" operation denotes choosing the corresponding token from $\mathbf{I}_n^{\mathrm{FC}_2} \in \mathbb{R}^{(T+1)\times D}$ so that $\mathbf{I}_n \in \mathbb{R}^D$. We followed ViT[7] to adopt layer normalization as the "Norm" operation.

After mining temporal heterogeneity information by TH-former, we obtained N lesions' features $\mathbf{I} = \{\mathbf{I}_n\} \in \mathbb{R}^{N\times D}$. Further, we used Object-Heterogeneity Transformer (OH-former) to model the relationships between different lesions. Its architecture was similar to that of the TH-former, as shown in Figure S4 C. Its input was $\hat{\mathbf{I}} = \{\mathbf{I}_0, \mathbf{I}_n\} \in \mathbb{R}^{(N+1)\times D}$ after adding a learnable distillation token. The lesion category was a discrete variable, and the offset days were a continuous variable. If we considered lesions' categories as position embedding in the transformer, it was hard to model some new categories of lesions in the actual clinical case. Therefore, we designed OH-former to accept only one input so that the model could dig out different lesions on its own and easily generalize to unknown lesions' categories. Specifically, the detailed process of OH-former was

$$\mathbf{S}^{\mathrm{MHA}_3} = \hat{\mathbf{I}} + \mathrm{Norm}\left(\mathrm{MHA}(\hat{\mathbf{I}})\right),$$

$$\mathbf{S}^{\mathrm{FC}_3} = \mathbf{S}^{\mathrm{MHA}_3} + \mathrm{Norm}\left(\mathbf{W}_{\mathrm{MHA}_3}^{\mathrm{T}} \mathbf{S}^{\mathrm{MHA}_3} + \mathbf{b}_{\mathrm{MHA}_3}\right),$$

$$\mathbf{S} = \mathrm{Distillation}(\mathbf{S}^{\mathrm{FC}_3}) \in \mathbb{R}^D.$$

The OH-former's output $\mathbf{S} \in \mathbb{R}^D$ was used as the input of a multi-layer perceptron (MLP), generating a risk score. The MLP comprised a layer normalization and two fully connected layers. The output of the first fully connected layer maintained D dimensions, while the output of the second one was two dimensions, corresponding to the high-risk and low-risk groups. We used the probability of the high-risk group as the predicted risk score.

Text S4. Details of TDLM.

The structure of TDLM was similar to that of LDLM, except that the CNN was replaced with a projection layer, as shown in Figure 1. The responsibility of the projection layer was to map consecutive numeric values into D dimension features in embedding spacing. For seven different types of tumor markers (LDH, NSE, CEA, CA125, CA199, CA724, and AFP), we separately used a fully connected layer as the projection layer. Like LDLM, TDLM used the TH-former to aggregate numerical information about tumor markers of the same category at different time points and simultaneously took the OH-former to aggregate the information of seven tumor markers. TDLM also used a multi-layer perceptron with the same structure as LDLM to predict risk scores.

Text S5. The details of missing data processing.

In the clinical scenario, it is difficult for every patient to have the same times of follow-up scans and examinations of tumor markers.

For time-series data with an object count of M, we used a vector $\mathbf{M} \in \{0,1\}^M$ to represent the absence of objects. If the object was missing, its corresponding position on $\mathbf{M}$ would be set to 0; otherwise, 1. We stacked $\mathbf{M}$ M times to get $\widehat{\mathbf{M}} = \{\mathbf{M}, \mathbf{M}, \cdots, \mathbf{M}\} \in \mathbb{R}^{M \times M}$, and the Equation S1 was rewritten as

$$\mathbf{F}_{out}^{(h)} = \mathrm{MHA}\left(\mathbf{F}_{in}^{(h)}\right) = \mathrm{Scaled}\left(\alpha\left(\mathbf{F}_{in}^{(h)}\right)\beta\left(\mathbf{F}_{in}^{(h)}\right)^T \odot \widehat{\mathbf{M}}\right)\gamma\left(\mathbf{F}_{in}^{(h)}\right) \in \mathbb{R}^{M \times D'},$$

where $\odot$ means element-wise multiplication. Replacing the attention calculation in the MHA modules in TH-former and OH-former with the aforementioned modifications enabled the model to cope with missing data.

Text S6

We used two types of loss functions for loss calculation. First, the patients in the training cohort were divided into a low-risk and high-risk groups based on the overall survival (OS) of 1 year (12 months). The model was trained to distinguish these two groups. We used cross-entropy loss (l_{ce}) for the loss function of this binary classification problem, expressed as

$$l_{ce} = -\sum_{i} y_i \log_2 h_\theta(x_i),$$

where x_i indicates the input data of ith patient, θ is the model's parameters, and $h_\theta(\cdot)$ represents the forward inference of the model. y_i indicates the group to which the ith patient belongs. If it is equal to 1, it means that it belongs to the high-risk group (OS$\leq$ 12 months), and if it is 0, it means that it belongs to the low-risk group (OS>12 months).

In addition, the rank relationship of OS among different patients was considered. For example, two patients, one with an OS of 50 months and another with an OS of 13 months, belonged to the low-risk group, but in fact, the risk score of the former should be lower than that of the latter. Therefore, inspired by the DeepSurv [3], we supervised the model to generate effective risk scores according to their overall survival by calculating the negative partial log-likelihood , called survival loss (l_{surv}), as follows:

$$l_{surv} = -\sum_{i,E_i=1}\left(h_\theta(x) - log\sum_{j\in\mathcal{R}(OS_i)} e^{h_\theta(x_j)}\right),$$

where $x = \{x_1, x_2, \cdots, x_B\}$ represents all patient samples in one mini-batch. $\mathcal{R}(OS_i)$ indicates the set of patients in this mini-batch whose overall survival exceeds the ith patient.

Based on the aforementioned description, the overall loss function was expressed as

$$l = l_{ce} + l_{surv}.$$

Previous studies used only the cross-entropy loss function [4; 5] or survival loss [3; 6] for training the prognostic model. The cross-entropy loss paid more attention to the discernible representations between different groups, and the survival loss was responsible for ordering the relationship between all samples, providing additional information for marginal samples. We verified through ablation experiments that aggregating both losses improved the model's performance. Defined as above, l_{surv} computed the relationship between satisfying samples within a mini-batch so that the mini-batch size affected the number of sample pairs for consideration. The performance of the model increased with the increase in mini-batch size, indicating that a larger mini-batch size could make the model stronger. We used a single RTX-3090 GPU device, and hence the maximum allowable mini-batch size was only 48. We speculate that potentially higher performance could be obtained using a larger mini-batch.

Text S7

We built our model using Python (version 3.7.7) and PyTorch (version 1.10.2 with cu113) [7]. ResNet18 was the default implementation of Torchvision (version 0.11.3 with cu113). The number of heads H for the MHA was set to 32. Besides, except for the last fully connected layer, the remaining fully connected layers were preceded by Dropout regularization [8] with a dropout probability of 0.5 to prevent overfitting. Moreover, we used a learning rate of 6e-4 for the AdamW [9] optimizer and set weight decay to 1e- 2. All models were trained with 30 epochs and adopted a batch size of 48. Moreover, we randomly initialized seeds for repeating the experiment five times to further ensure the reproducibility of our model and recorded their performances in Table S8.

Table S1. CT protocol of the four centers

Parameters	Center 1<br>Peking University Cancer Hospital & Institute	Center 2<br>The First Affiliated Hospital of Zhengzhou University	Center 3<br>Nanjing Drum Tower Hospital	Center 4<br>Ruijin Hospital
CT version	Spectral CT (Lightspeed 64 VCT and Discovery CT750 HD scanner, GE Healthcare, USA)	Spectral CT (Discovery CT750 HD scanner, GE Healthcare, USA)	uCT 780, United Imaging, Shanghai, China	Spectral CT (Discovery CT750 HD scanner, GE Healthcare, USA)<br>or dual-source CT (Siemens SOMATOM Definition Force, Siemens Healthineers, Erlangen, Germany)
CT tube voltage	120 kVp	120 kVp	120 kVp	80/140 kVp; 70/150 kVp
CT tube current	120–550 mA	120–550 mA	150–250 mA	120–230 mA
CT rotation time	0.76–0.80 s	0.50 s	0.7 s	0.50–0.80 s
CT detector collimation	64 × 0.625 mm	64 × 0.625 mm	64 × 0.625 mm	64 × 0.625 mm
Contrast agent type	Omnipaque, GE Healthcare, USA	Omnipaque, GE Healthcare, USA	Omnipaque, GE Healthcare, USA	Hengrui Pharmaceutical Co., Ltd., China
Contrast agent concentration	300 mgI/mL	350 mgI/mL	350 mgI/mL	350 mgI/mL
Contrast agent dosage	Infused 2 mL/kg body weight	Infused 1.5 mL/kg body weight	Infused 1.5 mL/kg body weight	Infused 1.5 mL/kg body weight
Contrast agent infused rate	3.5 mL/s	3.0 mL/s	3.0 mL/s	3.0–3.5 mL/s
Arterial-phase interval time	35–40 s after injecting the contrast agent	30 s after injecting the contrast agent	40 s after injecting the contrast agent	35–40 s after injecting the contrast agent
Venous-phase interval time	70 s after injecting the contrast agent	70 s after injecting the contrast agent	70 s after injecting the contrast agent	70 s after injecting the contrast agent
Image matrix	512 × 512	512 × 512	512 × 512	512 × 512
Field of view	500 × 500 mm	500 × 500 mm	500 × 500 mm	500 × 500 mm
Reconstruction image thickness	5 mm	5 mm	5 mm	5 mm

Table S2. Normalization parameters for different centers

Mean [±SD]	Center 1<br>Peking University Cancer Hospital & Institute		Center 2<br>The First Affiliated Hospital of Zhengzhou University		Center 3<br>Nanjing Drum Tower Hospital		Center 4<br>Ruijin Hospital	
	Venous phase							
Stomach	0.254	[±0.273]	0.350	[±0.271]	0.309	[±0.293]	0.361	[±0.263]
Lymph node	0.401	[±0.222]	0.439	[±0.234]	0.602	[±0.231]	0.416	[±0.247]
Liver	0.539	[±0.220]	0.601	[±0.137]	0.650	[±0.061]	0.515	[±0.217]
Adrenal gland	0.466	[±0.219]						
Peritoneum	0.348	[±0.248]						
Bone	0.746	[±0.300]						
Soft tissue	0.303	[±0.233]						
Spleen	0.474	[±0.286]						
Lung								
Other	0.342	[±0.301]						
	Arterial phase							
Stomach	0.250	[±0.273]	0.381	[±0.253]	0.280	[±0.266]	0.346	[±0.262]
Lymph node	0.395	[±0.232]	0.448	[±0.228]	0.515	[±0.259]	0.360	[±0.264]
Liver	0.489	[±0.210]	0.547	[±0.182]			0.470	[±0.208]
Adrenal gland	0.436	[±0.238]						
Peritoneum	0.343	[±0.245]						
Bone	0.631	[±0.379]						
Soft tissue	0.427	[±0.243]						
Spleen	0.609	[±0.111]						
Lung	0.190	[±0.224]					0.248	[±0.281]
Other								

Table S3. Normalization parameters for different tumor markers

	LDH	NSE	CEA	CA125	CA19-9	CA724	AFP
Mean	247.593	210.247	40.859	762.060	92.167	30.695	15.677
SD	202.593	2294.276	120.945	4093.535	607.069	188.111	11.749

AFP, Alpha-fetoprotein; CA125, carbohydrate antigen 125; CA19-9, carbohydrate antigen 19-9; CA724, carbohydrate antigen 724; CEA, carcinoembryonic antigen; LDH, lactate dehydrogenase; NSE, neuron-specific enolase

Table S4. Statistics of annotation information.

n (%) [±SD]	Total		Training cohort		Internal validation cohort		External validation cohort		Prospective cohort	
**Bounding boxes**										
Total number [*n* (%)]	4104		2125		728		682		569	
Base Line (BL)	1278	(31.14)	561	(26.40)	271	(37.23)	1278	(31.14)	561	(26.40)
First follow-up (1F)	1259	(30.68)	559	(26.31)	265	(36.40)	1259	(30.68)	559	(26.31)
Second follow-up (2F)	1024	(24.95)	462	(21.74)	192	(26.37)	1024	(24.95)	462	(21.74)
Third follow-up (3F)	333	(8.11)	333	(15.67)						
Fourth follow-up (4F)	210	(5.12)	210	(9.88)						
**Area (mm^2)**						***p*-value**		***p*-value**		***p*-value**
p-value*						**0.600**		**0.893**		**0.116**
Total	454.35	[±973.21]	422.51	[±1042.30]	443.72	[±794.32]	453.57	[±739.23]	638.53	[±1174.68]
Lymph node	304.91	[±457.73]	305.67	[±503.76]	271.12	[±309.13]	341.96	[±538.64]	320.44	[±334.77]
Liver	641.47	[±1359.58]	593.63	[±1521.36]	555.29	[±735.90]	628.15	[±954.07]	1220.71	[±1870.64]
Aden	522.47	[±905.16]	445.91	[±939.53]	533.06	[±165.49]	275.03	[±143.36]	970.89	[±1361.05]
Peritoneum	788.02	[±1631.84]	691.18	[±1376.56]	1589.16	[±2421.49]	160.48	[±109.79]	224.91	[±169.64]
Bone	75.55	[±67.76]	75.55	[±67.76]						
Soft tissue	611.52	[±498.24]	831.42	[±420.86]	61.77	[±54.03]				
Spleen	702.76	[±507.32]	162.63	[±31.59]					1062.85	[±322.70]
Lung	264.77	[±280.95]	145.06	[±119.96]	294.20	[±150.79]	367.92	[±163.70]	480.49	[±462.99]
Other	2470.00	[±2407.28]			2558.51	[±2659.93]			2366.74	[±2069.19]
Short diameter (mm)										
Total	15.57	[±12.65]	14.94	[±12.75]	14.98	[±10.81]	16.25	[±12.05]	18.96	[±15.43]
Lymph node	13.45	[±8.66]	13.51	[±9.23]	12.34	[±7.46]	14.06	[±8.45]	14.57	[±8.04]
Liver	18.86	[±16.10]	17.36	[±16.14]	18.87	[±12.71]	19.88	[±15.42]	27.14	[±21.37]
Aden	16.45	[±14.12]	15.22	[±14.30]	15.33	[±5.31]	13.23	[±4.61]	24.46	[±20.85]
Peritoneum	15.33	[±14.09]	17.53	[±16.80]	15.41	[±13.99]	9.30	[±4.25]	12.55	[±6.30]
Bone	6.39	[±3.58]	6.39	[±3.58]						
Soft tissue	21.62	[±12.24]	28.46	[±6.53]	4.51	[±2.74]				
Spleen	26.34	[±13.00]	11.65	[±2.40]					36.13	[±6.18]
Lung	8.55	[±4.30]	6.58	[±3.88]	10.57	[±1.83]	9.80	[±3.09]	11.45	[±4.46]
Other	43.00	[±22.47]			44.41	[±23.45]			41.34	[±21.14]
Long diameter (mm)										
Total	23.52	[±17.71]	22.27	[±16.56]	24.87	[±20.87]	24.02	[±15.14]	26.54	[±19.27]
Lymph node	21.16	[±11.75]	21.09	[±12.06]	20.27	[±11.20]	22.66	[±12.21]	21.35	[±10.35]
Liver	25.99	[±20.84]	23.99	[±21.28]	26.89	[±16.95]	26.37	[±18.65]	36.90	[±26.65]
Aden	28.88	[±16.71]	24.35	[±15.34]	42.99	[±7.37]	25.38	[±6.81]	31.61	[±23.64]
Peritoneum	33.47	[±41.70]	28.82	[±23.31]	57.83	[±69.62]	18.42	[±8.39]	18.75	[±7.58]
Bone	10.78	[±4.60]	10.78	[±4.60]						
Soft tissue	28.77	[±12.65]	35.31	[±7.98]	12.43	[±5.22]				
Spleen	31.34	[±12.91]	16.83	[±1.88]					41.02	[±6.44]
Lung	13.94	[±5.82]	13.55	[±6.17]	16.69	[±3.81]	12.18	[±3.56]	14.30	[±6.41]
Other	60.44	[±29.73]			60.06	[±30.82]			60.88	[±28.40]

*Wilcoxon signed-rank test, Bonferroni correction.

Table S5. Ablation study for lesions input to the model

Lesions	C-index (95% CI)	HR (95% CI)	HR (*p*-value)	AUC (95% CI)
Primary tumor	0.581 (0.460–0.702)	1.639 (0.288–9.322)	0.5772	0.667 (0.461–0.873)
Target lesions	0.683 (0.583–0.783)	7.485 (2.156–25.987)	0.0015	0.804 (0.662–0.945)
Primary + Target	0.725 (0.614–0.836)	25.111 (4.535–139.034)	0.0002	0.844 (0.702–0.986)

AUC, area under the curve; C-index, concordance index; HR, hazard ratio.

Table S6. Ablation study for temporal position embedding on the validation cohort

	C-index (95% CI)	HR (95% CI)	HR (*p*-value)	AUC (95% CI)
w/o TPE	0.679 (0.538–0.820)	32.414 (4.147–253.37)	0.0009	0.689 (0.474–0.903)
w/ TPE	0.725 (0.614–0.836)	25.111 (4.535–139.034)	0.0002	0.844 (0.702–0.986)

TPE, Temporal position embedding; AUC, area under the curve; C-index, concordance index; HR, hazard ratio.

Table S7. Ablation study for different loss functions on the validation cohort

Loss function	C-index (95% CI)	HR (95% CI)	HR (*p*-value)	AUC (95% CI)
l_{ce}	0.701 (0.594–0.808)	17.521 (2.804–109.466)	0.0022	0.856 (0.708–1.003)
l_{surv}	0.686 (0.585–0.786)	7.403 (2.071–26.465)	0.0021	0.851 (0.729–0.972)
$l_{ce} + l_{surv}$	0.725 (0.614–0.836)	25.111 (4.535–139.034)	0.0002	0.844 (0.702–0.986)

l_{ce}, Cross-entropy loss. AUC, area under the curve; C-index, concordance index; HR, hazard ratio. l_{surv}, Survival loss.

Table S8. Impact of mini-batch size for survival loss on the validation cohort

Mini-batch size	C-index (95% CI)	HR (95% CI)	HR (*p*-value)	AUC (95% CI)
4	0.715 (0.601–0.828)	88.052 (5.415–1431.745)	0.0016	0.875 (0.733–1.017)
16	0.724 (0.623–0.824)	19.759 (2.500–156.197)	0.0003	0.836 (0.694–0.978)
48	0.725 (0.614–0.836)	25.111 (4.535–139.034)	0.0002	0.844 (0.702–0.986)

AUC, area under the curve; C-index, concordance index; HR, hazard ratio.

Table S9. Reproducibility analysis of different random seeds on the validation cohort

Seed	C-index (95% CI)	HR (95% CI)	HR (*p*-value)	AUC (95% CI)
0	0.725 (0.614–0.836)	25.111 (4.535–139.034)	0.0002	0.844 (0.702–0.986)
1	0.752 (0.658–0.846)	53.520 (7.206–397.521)	0.0001	0.906 (0.807–1.005)
2	0.719 (0.630–0.808)	40.210 (5.598–288.825)	0.0002	0.865 (0.739–0.991)
3	0.737 (0.638–0.836)	58.794 (6.220–555.756)	0.0004	0.914 (0.819–1.009)
4	0.707 (0.613–0.802)	14.717 (3.477–62.295)	0.0003	0.869 (0.742–0.996)
Mean ± SD	0.728 ± 0.015	38.470 ± 16.645	0.0002 ± 0.0001	0.880 ± 0.026

AUC, Area under the curve; C-index, concordance index; HR, hazard ratio; SD, standard deviation.

Table S10. Performance comparisons of all models in predicting overall survival and AUCs in predicting one year survival in four cohorts.

Models	C-index (95% CI)	HR (95% CI)	HR (*p*-value)	AUC (95% CI)
Training cohort (*n* = 91)				
RECIST	0.648 (0.572–0.717)	1.858 (1.363–2.534)	<0.0001	0.753 (0.628–0.860)
TB-Δ	0.613 (0.482–0.696)	1.006 (1.000–1.013)	0.0447	0.675 (0.514–0.788)
LDLM-BS	0.661 (0.586–0.725)	4.715 (1.939–11.466)	0.0006	0.744 (0.606–0.865)
LDLM-1F	0.721 (0.652–0.787)	5.573 (2.834–10.958)	<0.0001	0.771 (0.651–0.877)
LDLM-2F	0.775 (0.712–0.829)	25.409 (9.676–66.725)	<0.0001	0.879 (0.775–0.961)
TDLM	0.717 (0.641–0.793)	9.230 (3.825–22.274)	<0.0001	0.780 (0.658–0.885)
Nomo-w/o TDLM	0.795 (0.736–0.851)	73.613 (24.399–222.092)	<0.0001	**0.903** (0.810–0.976)
Nomo-LDLM-2F	**0.807** (0.743–0.865)	115.751 (34.611–387.108)	<0.0001	0.891 (0.785–0.967)
Internal validation cohort (*n* = 46)				
RECIST	0.652 (0.523–0.771)	1.583 (1.100–2.277)	0.0133	0.759 (0.572–0.937)
TB-Δ	0.594 (0.483–0.724)	1.011 (1.000–1.022)	0.0456	0.651 (0.437–0.862)
LDLM-BS	0.632 (0.498–0.768)	3.225 (0.993–10.473)	0.0513	0.701 (0.519–0.868)
LDLM-1F	0.721 (0.602–0.830)	8.958 (2.525–31.780)	0.0007	0.838 (0.673–0.959)
LDLM-2F	0.725 (0.601–0.836)	25.111 (4.535–139.034)	0.0002	0.844 (0.673–0.971)
TDLM	0.718 (0.589–0.829)	15.172 (2.527–91.080)	0.0029	0.867 (0.739–0.961)
Nomo-w/o TDLM	0.736 (0.595–0.865)	31.678 (6.390–157.050)	<0.0001	0.836 (0.645–1.000)
Nomo-LDLM-2F	**0.752** (0.635–0.871)	23.911 (5.229–109.337)	<0.0001	**0.894** (0.728–1.000)
External validation cohort (*n* = 37)				
RECIST	0.627 (0.516–0.753)	2.125 (1.147–3.937)	0.0166	0.653 (0.396–0.930)
TB-Δ	0.527 (0.407–0.788)	1.001 (0.993–1.010)	0.7578	0.516 (0.342–0.896)
LDLM-BS	0.619 (0.463–0.756)	5.232 (0.823–33.237)	0.0794	0.583 (0.290–0.827)
LDLM-1F	0.632 (0.467–0.797)	5.132 (0.361–72.99)	0.2272	0.696 (0.373–0.966)
LDLM-2F	0.669 (0.503–0.836)	10.553 (0.713–156.072)	0.0865	0.683 (0.350–0.957)
Nomo-w/o TDLM	**0.709** (0.562–0.855)	13.837 (1.846–103.746)	0.0106	**0.771** (0.510–1.000)
Prospective cohort (*n* = 33)				
RECIST	0.644 (0.440–0.783)	1.343 (0.880–2.048)	0.1710	0.770 (0.547–0.959)
TB-Δ	0.630 (0.446–0.768)	1.007 (1.001–1.012)	0.0226	0.738 (0.427–0.965)
LDLM-BS	0.524 (0.466–0.705)	1.499 (0.229–9.795)	0.6724	0.472 (0.218–0.725)
LDLM-1F	0.630 (0.484–0.809)	2.983 (0.598–14.875)	0.1824	0.536 (0.241–0.832)
LDLM-2F	0.726 (0.566–0.877)	24.972 (2.608–239.116)	0.0052	0.690 (0.419–0.962)
TDLM	0.595 (0.475–0.743)	2.666 (0.354–20.068)	0.3411	0.678 (0.440–0.915)
Nomo-w/o TDLM	**0.758** (0.594–0.892)	15.718 (2.481–99.578)	0.0034	0.803 (0.554–1.000)
Nomo-LDLM-2F	0.741 (0.570–0.882)	8.809 (1.701–45.624)	0.0095	**0.809** (0.561–1.000)

AUC, Area under the curve; C-index, concordance index; HR, hazard ratio.

Figure S1. A sample for the preprocessing of CT images. Each slice image underwent preprocessing such as cropping, padding, scaling, and normalization. Furthermore, this slice's upper and lower slices were considered to form a three-channel image as CNN's inputs, thereby enhancing contextual information. For the baseline image, the radiologist annotated the location and the size of the lesion (as shown in a red bounding box in the first row). For the follow-up images, the locations were also annotated (as shown in red bounding boxes in the second and third rows), and the size information of the corresponding lesion at the baseline was used for preprocessing.

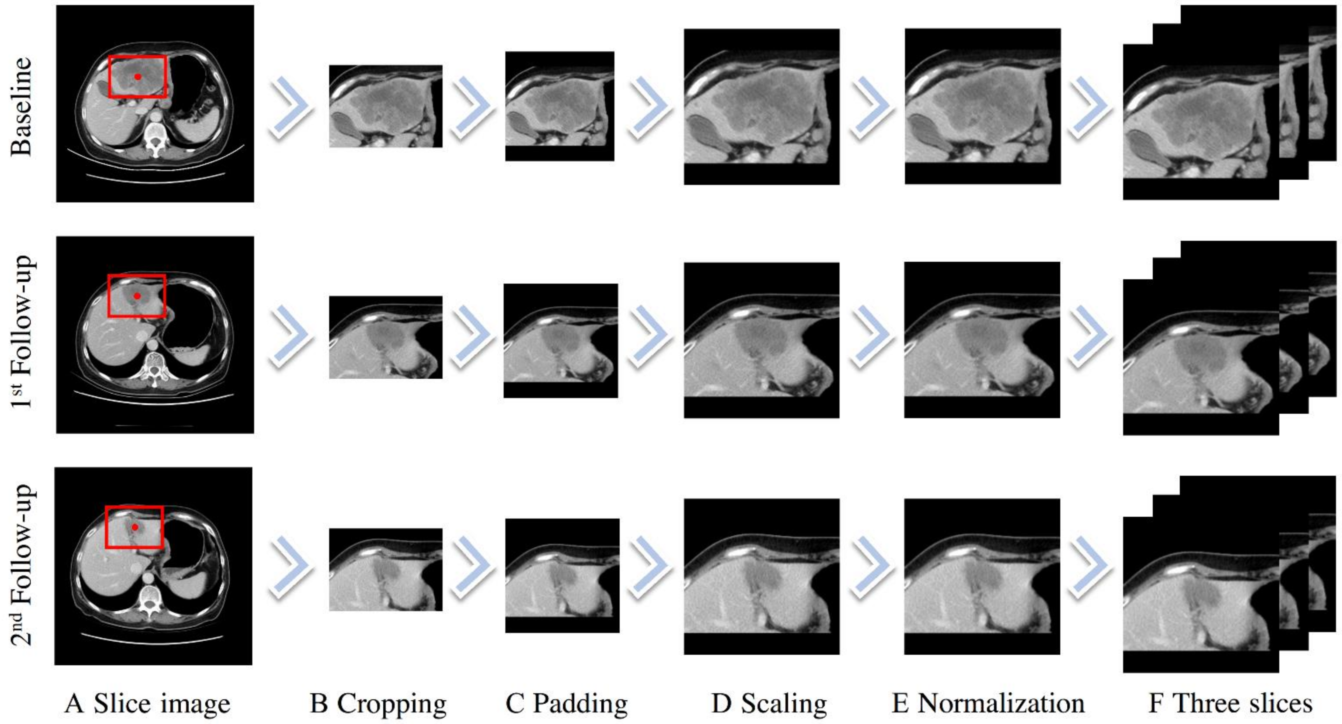

Figure S2. A typical example for input, including bounding boxes at baseline and at the first two follow-up visits. The example pictures include primary tumor (GC), lymph nodes, liver, and soft tissue metastases. We improved the robustness of the model by randomly rotating the input ROI image by –30 degrees to 30 degrees.

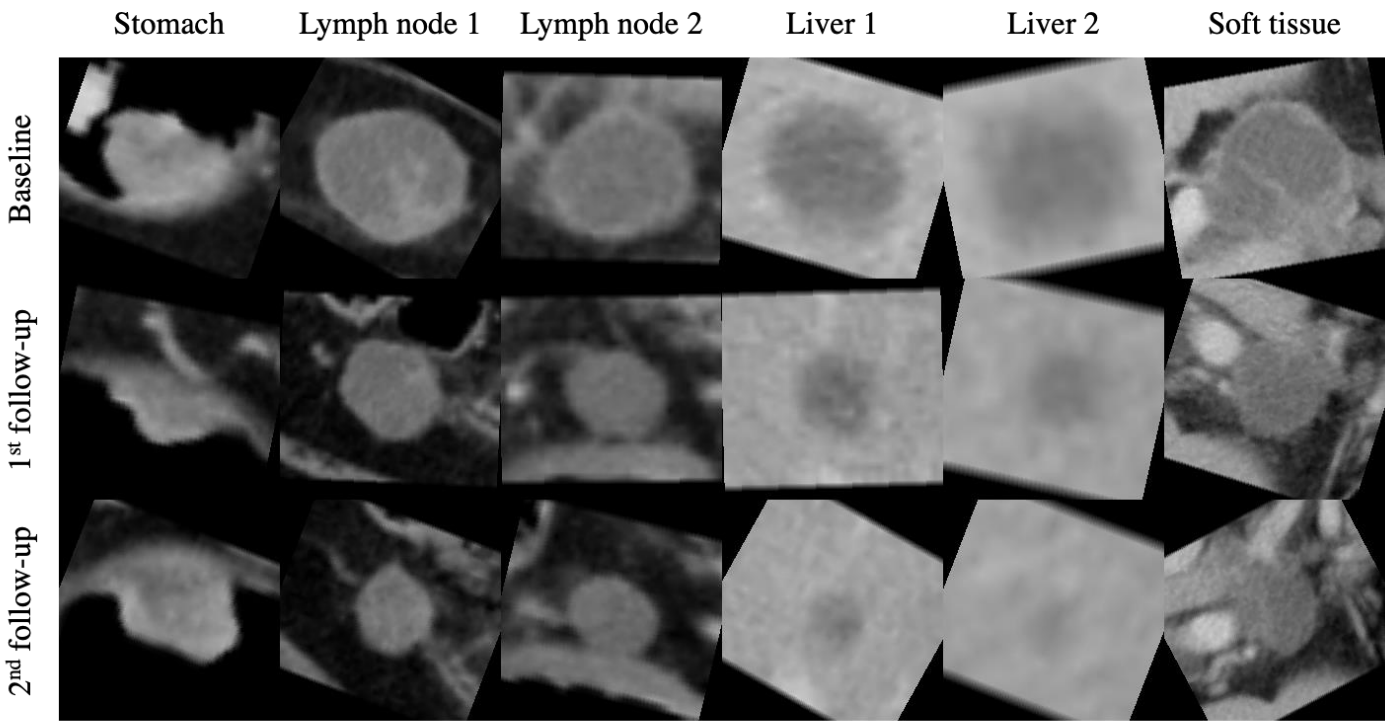

Figure S3. Architecture of CNN-based feature extractor based on ResNet-18. "/2" indicates that the convolution or pooling operation had a stride of 2. Each two consecutive convolutional layers in blue and the residual path formed a Basic Block. The red arrow indicates which output was used as the feature in this study. The dotted arrow indicates that the residual path contained an additional convolutional layer with a stride of 2 to ensure that the input and output feature maps of the "Basic Block" were of the same resolution so that they could be fused.

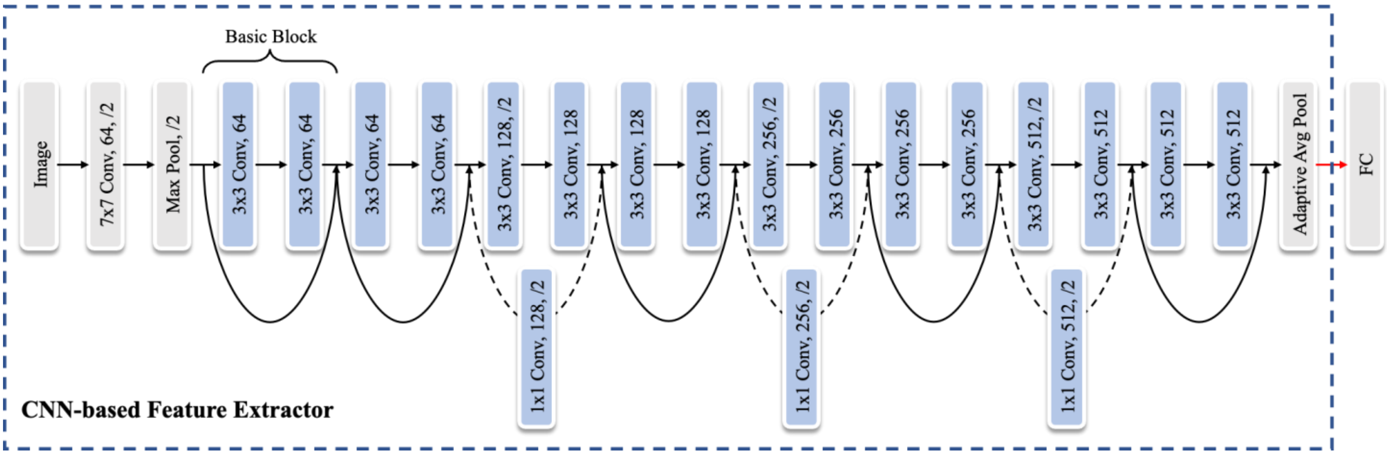

Figure S4. Details of modules. **A** is the architecture of the multi-head attention module (MHA), illustrating the process of grouping and aggregating at the channel level for different heads. **B** shows the proposed time-heterogeneity transformer, called TH-former, which stacked two stages. Each stage consisted of an MHA module followed by a layer normalization layer with a residual path. A fully connected layer followed a layer normalization layer with a residual path, also called a forward feedback network (FFN). Different information at different time points was aggregated into the distillation token. The “Distillation” operation was to choose the corresponding token in the results. **C** shows the proposed object-heterogeneity transformer, called OH-former, which only used a stage for aggregating the information of different objects.

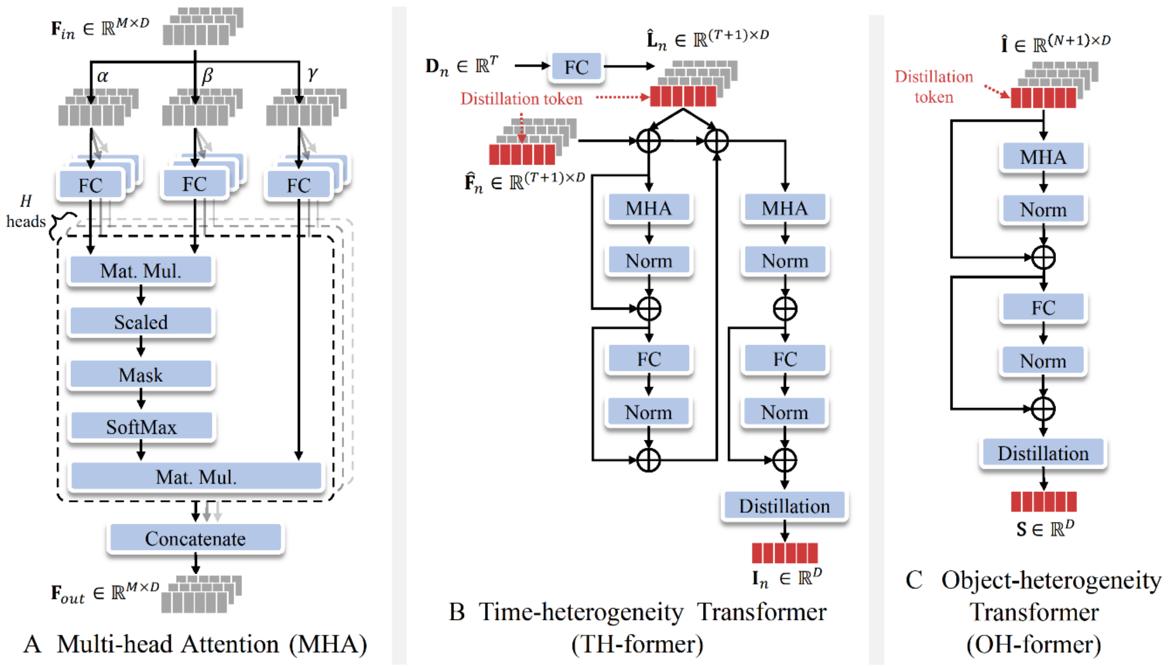

Figure S5. Through an attention-weighting mechanism, Time-heterogeneity Transformer and Object-heterogeneity Transformer combined different lesion features from a patient at different time points to generate Lesion-based Deep Learning scores.

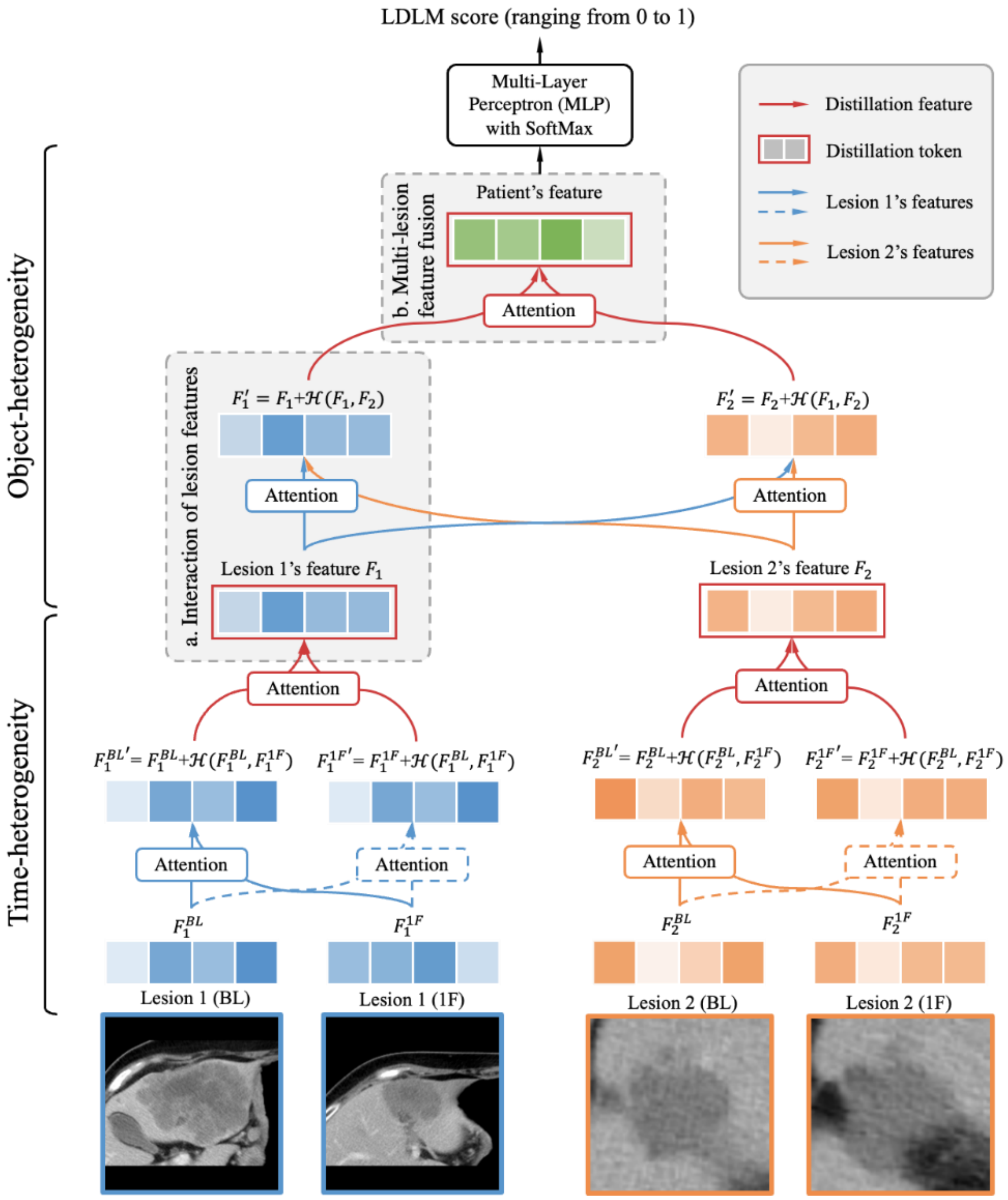

Figure S6. Overall survival analysis for different centers and cohorts.

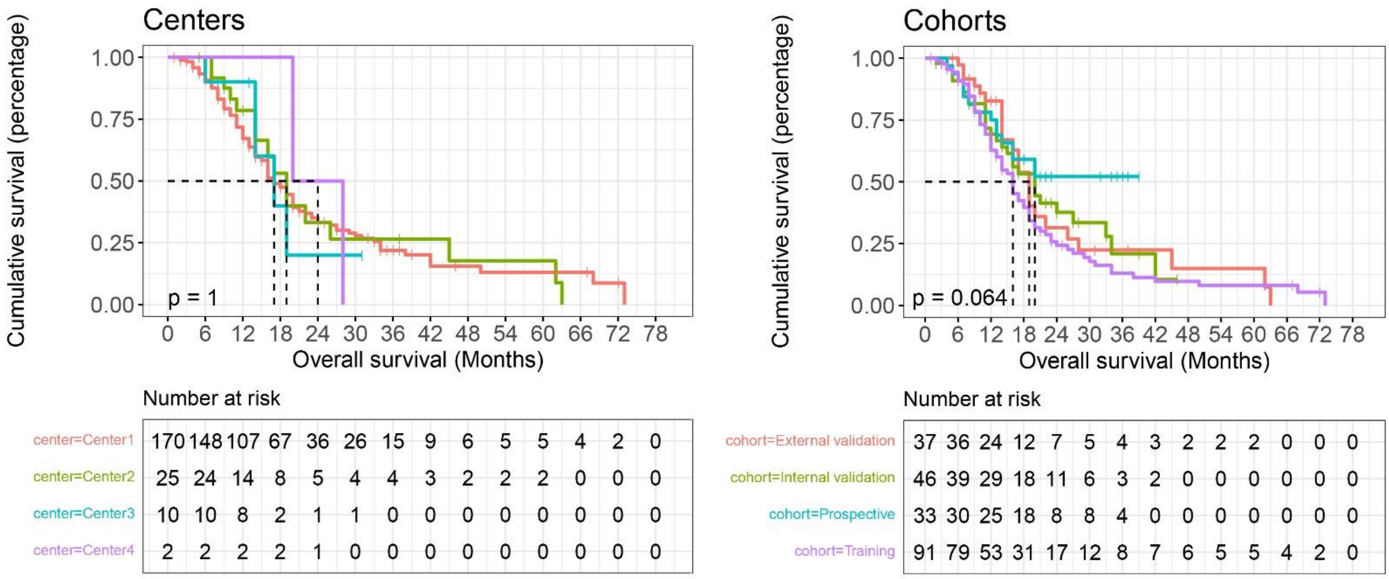

Figure S7. Developed nomogram without TDLM (Nomo-w/o TDLM). The nomogram was built in the training cohort, incorporating the LDLM score, RECIST, sex, and HER2 status (**A**). Calibration curve of the Nomo-w/o TDLM in the training, internal validation, external validation, and prospective cohorts (**B**).

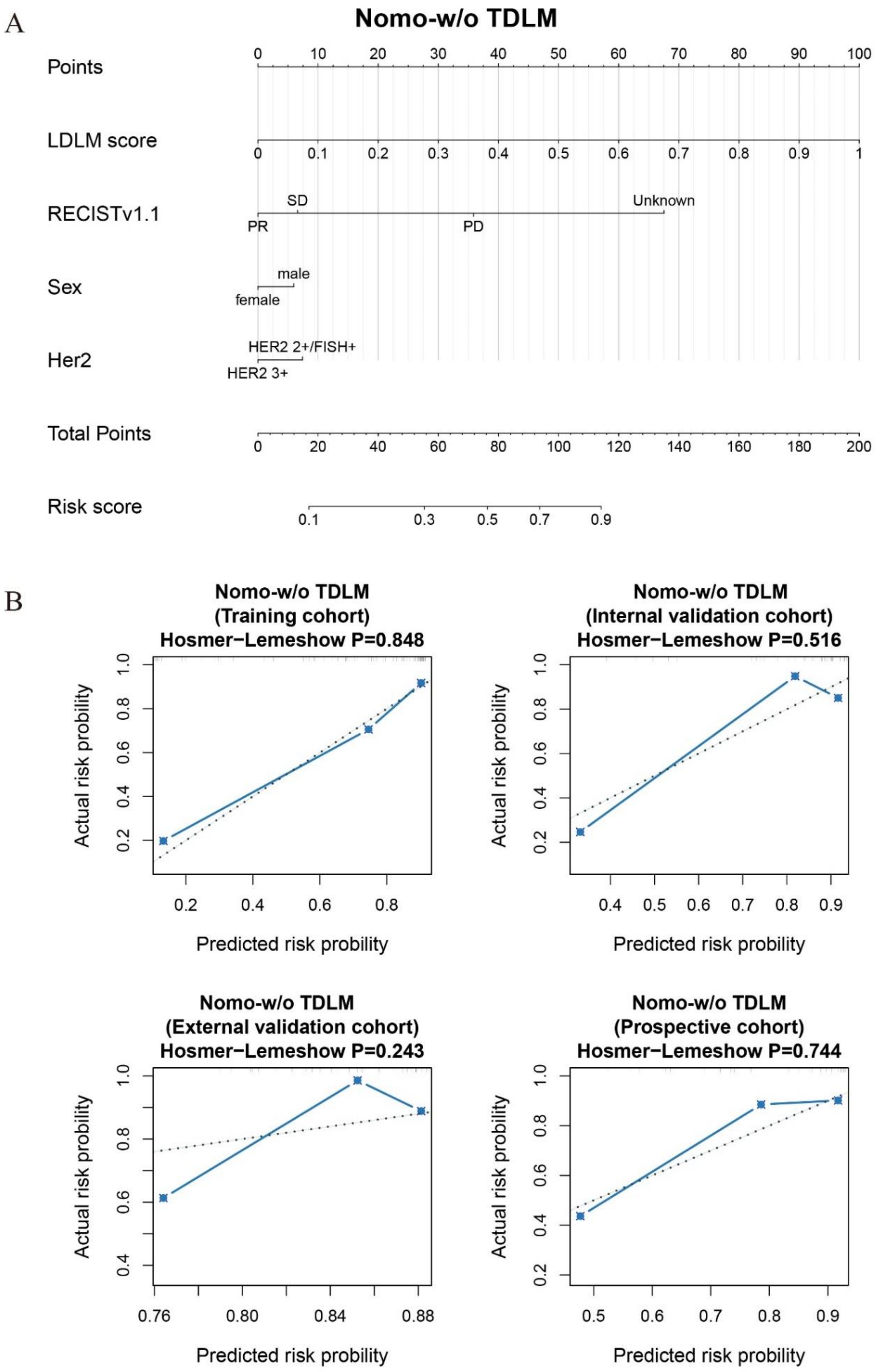

Figure S8. Overall survival Kaplan–Meier analysis was performed in the training, internal validation, external test, and prospective cohorts stratified by RECIST 1.1 ($p < 0.05$, log-rank test).

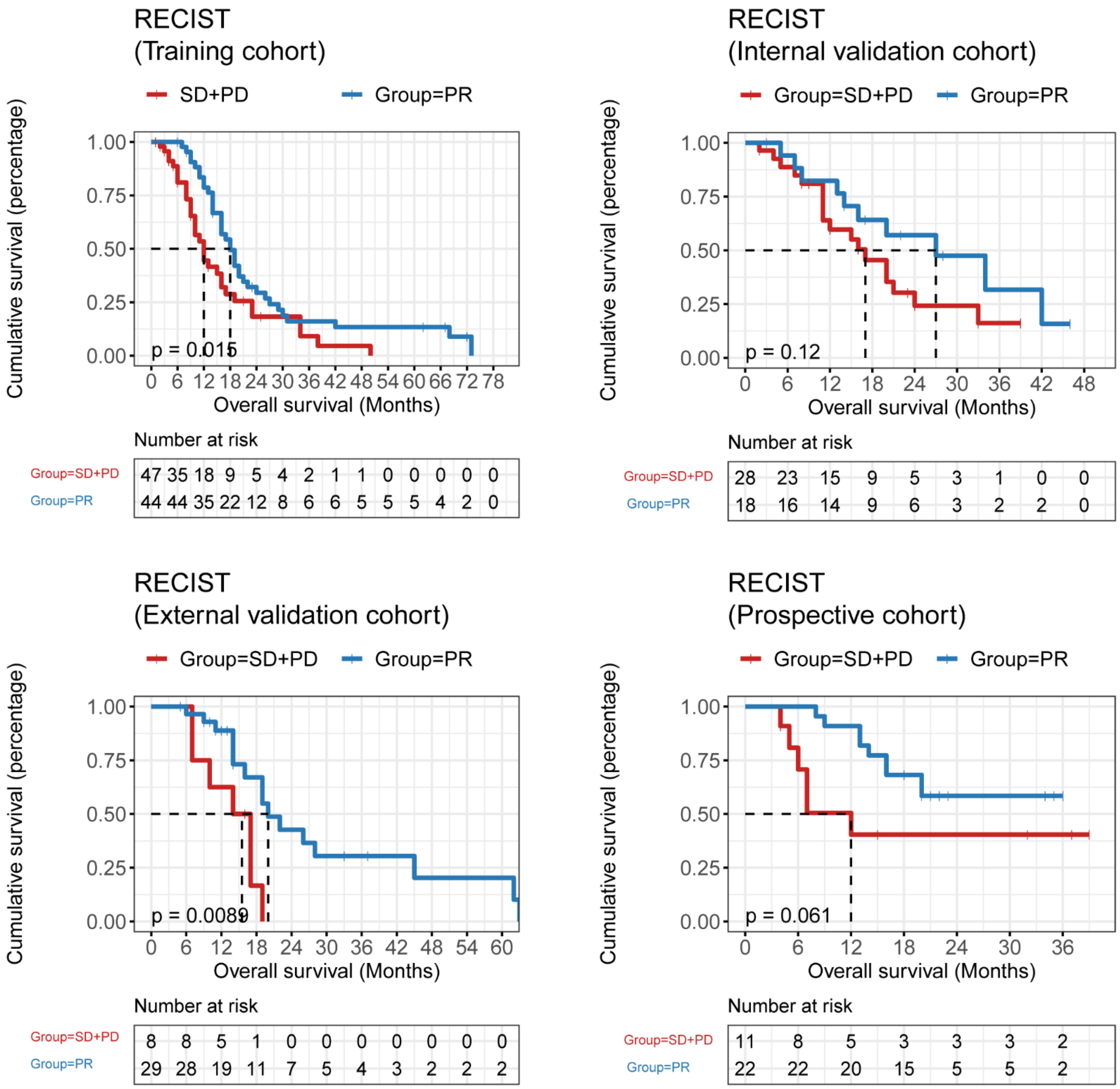

Figure S9. A sample case used to illustrate how the masking operation coped with the absence of certain tumor markers. Suppose that three tumor markers, NSE, CA199 and CA724, were absent from the sample data (shown in dark gray), the importance scores of the corresponding positions in the attention matrix (7×7) among the seven markers were set to 0. Then, these missing tumor markers were aggregated to the final feature vector with a weight of 0 (shown in gray in the formula calculation part of the figure).

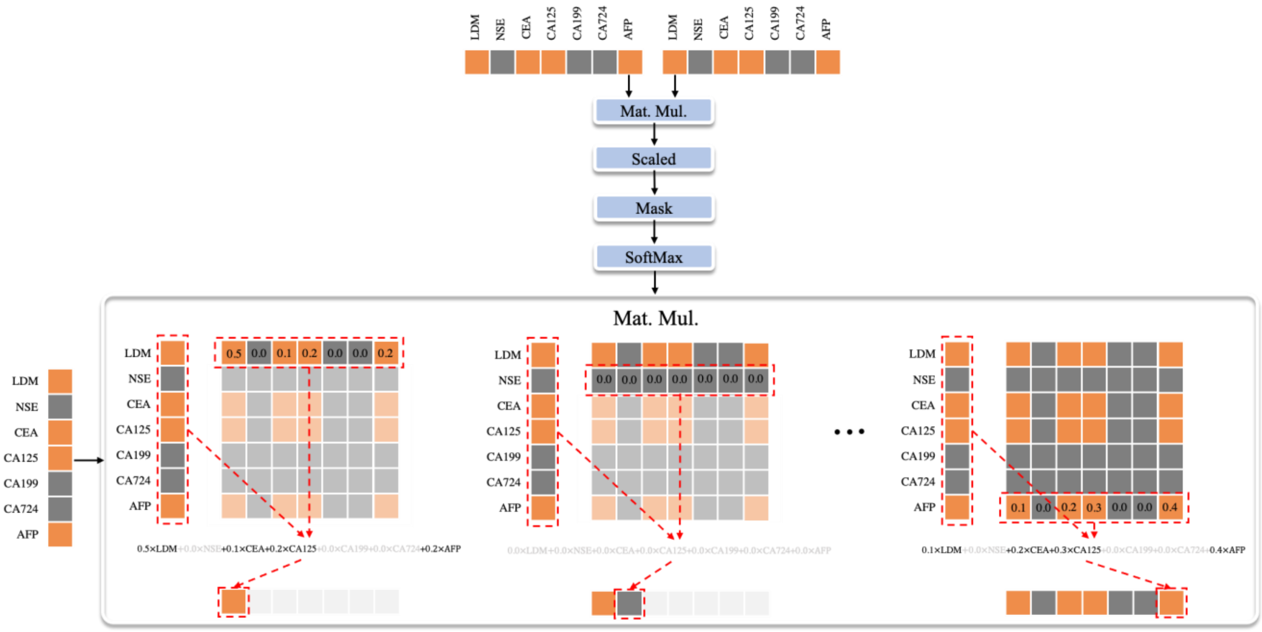

References

1 Guo X, Yang C, Li B, Yuan Y (2021) Metacorrection: Domain-aware meta loss correction for unsupervised domain adaptation in semantic segmentationProceedings of the IEEE/CVF Conference on Computer Vision and Pattern Recognition, pp 3927-3936

2 He K, Zhang X, Ren S, Sun J (2016) Deep residual learning for image recognitionProceedings of the IEEE conference on computer vision and pattern recognition, pp 770-778

3 Katzman JL, Shaham U, Cloninger A, Bates J, Jiang T, Kluger Y (2018) DeepSurv: personalized treatment recommender system using a Cox proportional hazards deep neural network. BMC Medical Research Methodology 18:24

4 Zhong J, Zhang C, Hu Y et al (2022) Automated prediction of the neoadjuvant chemotherapy response in osteosarcoma with deep learning and an MRI-based radiomics nomogram. Eur Radiol 32:6196-6206

5 Feng B, Chen X, Chen Y et al (2020) Solitary solid pulmonary nodules: a CT-based deep learning nomogram helps differentiate tuberculosis granulomas from lung adenocarcinomas. Eur Radiol 30:6497-6507

6 Mobadersany P, Yousefi S, Amgad M et al (2018) Predicting cancer outcomes from histology and genomics using convolutional networks. Proc Natl Acad Sci U S A 115:E2970-e2979

7 Paszke A, Gross S, Massa F et al (2019) PyTorch: An Imperative Style, High-Performance Deep Learning Library.

8 Srivastava N, Hinton G, Krizhevsky A, Sutskever I, Salakhutdinov R (2014) Dropout: a simple way to prevent neural networks from overfitting. The journal of machine learning research 15:1929-1958

9 Loshchilov I, Hutter F (2018) Fixing weight decay regularization in adam.
